# Radiocesium-bearing microparticles discovered on masks worn during indoor cleaning

**DOI:** 10.1038/s41598-023-37191-0

**Published:** 2023-06-20

**Authors:** Shogo Higaki, Hiroko Yoshida-Ohuchi, Naohide Shinohara

**Affiliations:** 1 Isotope Science Center, The University of Tokyo, 2-11-16 Yayoi, Bunkyo-ku, Tokyo, 113-0032 Japan; 2 Cyclotron and Radioisotope Center, Tohoku University, Sendai, Japan; 3 National Institute of Advanced Industrial Science and Technology, Tsukuba, Japan

**Keywords:** Environmental sciences, Risk factors

## Abstract

A decade has passed since the Fukushima Dai-ichi Nuclear Power Plant (FDNPP) accident on March 11, 2011. However, radioactive particles have recently been detected in the indoor air of some residences near the FDNPP. Following the recommendations of previous research, we determined the presence of radiocesium-bearing microparticles (CsMPs) and measured the radioactivity of radiocesium that adhered on non-woven face masks worn by six persons during the indoor cleaning of 59 residences in Namie, Futaba, Okuma, and Tomioka towns in Fukushima Prefecture. Of the 284 masks worn in this study, significant ^137^Cs radioactivity was detected in 268, and 44 new CsMPs were discovered in 28. The results of this study also suggest the presence of highly concentrated soluble radiocesium particles or soluble radioactive cesium aerosols adhered to house dust. This implies that the CsMPs constituted a large proportion of radioactivity in the indoor air contamination for particles in the 1.0–2.5 µm size range due to the radioactive radiocesium particles. It is desirable to wear masks during cleaning to prevent inhalation of CsMPs.

## Introduction

The release of large amounts of radioactive materials from the Fukushima Dai-ichi Nuclear Power Plant (FDNPP) accident—caused by the tsunami that occurred after the Great East Japan Earthquake on March 11, 2011—forced the inhabitants of the towns and villages surrounding the FDNPP, including Futaba, Okuma, Namie, and Tomioka towns and Katsurao and Iitate villages, to evacuate. The effects of radioactive materials with relatively short physical half-lives, such as ^132^Te and ^131^I, dissipate within a short period. However, contamination caused by radiocesium has persistent long-term effects, owing to its chemical properties and relatively long half-life. Since April 2012, the Japanese government has designated areas around the FDNPP as difficult-to-return, restricted residence, restricted evacuation, return, and evacuation order cancellation preparation zones^1^. Evacuation orders have gradually been lifted over time. For instance, all evacuation orders for the restricted residence and evacuation order cancellation preparation zones were lifted in March 2020—nine years after the accident^2^. In August 2022, the specified reconstruction base located in the difficult-to-return zone in Futaba town was lifted; after 11 years, the uninhabited municipalities in the town were eliminated. In April 2023, the specified reconstruction base in Tomioka town was lifted. However, not every evacuation zone has been lifted. Some residents intend to return to their homes and permanently live there; thus, information on indoor deposition of radioactive materials should be gathered to assess the effects of the nuclear accident on their health. To educate the residents on health risks associated with radioactive materials, a research campaign on radiocesium contamination in evacuation zones near the FDNPP was conducted from 2016 to 2019; some findings have already been published.

Shinohara and Yoshida-Ohuchi^3^ reported ^137^Cs radioactivity in house dust sampled from 21 wooden buildings, based on house dust particle size. They observed that the ^137^Cs radioactivity concentration in house dust increased as particle size decreased; the concentration of ^137^Cs in house dust with particle size < 4–1000 μm was inversely related to the square of the distance of the house from the FDNPP. When 60 residences were cleaned in 2019, ^137^Cs radioactivity was detected in the indoor air of these residences^4^. The concentration of radiocesium radioactivity in the indoor air decreased as the aerodynamic diameter of aerosol particles decreased; it was also inversely proportional to the square of the distance from the FDNPP. In 2020, ^137^Cs radioactivity was detected in 65 houses and buildings 1.6–16.1 km away from the FDNPP; these residences were being vacuumed and dusted at the time of inspection^5^. This study estimated internal exposure from the ingestion of house dust and inhalation of aerosols.

Soluble sulfate aerosols^6^ and insoluble radiocesium-bearing microparticles (CsMPs)^7^ are forms of radiocesium that have been identified in the environment following the nuclear accident. However, it has been shown that CsMPs do not account for all the radioactive cesium in a plume^8^. Since radiocesium causes significant internal exposure through inhalation, CsMPs may also cause internal exposure through inhalation. During the research campaign, the presence of CsMPs and the amount of radiocesium that adhered to the masks worn by persons were determined. At the height of the COVID-19 pandemic, face masks were widely used to prevent inhalation of air-borne pathogens. Most Japanese have been wearing masks because they believe in their significance and efficacy. In a previous study, 14 masks worn by three persons in six residences in Okuma and Futaba towns were examined; the presence of non-spherical, large, insoluble particles originating from Unit 2 of FDNPP was reported for the first time^9^. Furthermore, the presence of CsMPs on the masks worn by the inhabitants of Fukushima Prefecture in the spring of 2013 was reported^10^.

In residences surrounding the FDNPP where residents were evacuated, the plume caused by the accident was confined indoors and was not considerably affected by weathering. Therefore, following the recommendations of a previous study^9^, we measured the amount of radiocesium that adhered to the non-woven fabric masks worn by persons while cleaning the indoor areas of the residences surrounding the FDNPP to determine the presence of CsMPs. The findings will be used in developing countermeasures to minimize the risks of inhaling radioactive particles.

## Materials and methods

### Locations of mask-wearing

**Figure 1:**
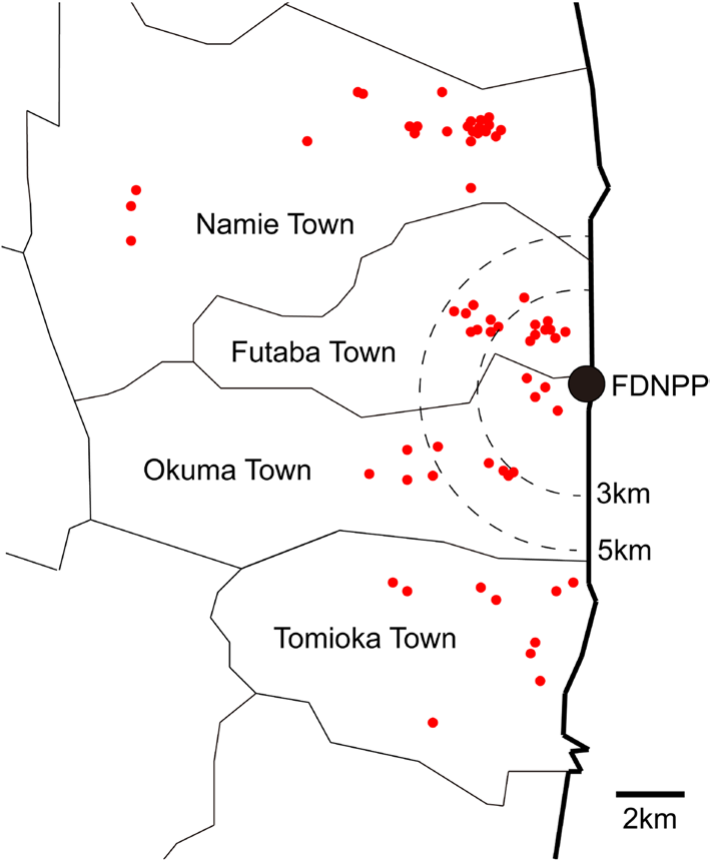
Map showing location of residence, created using Adobe Illustrator software (version 27.5).

Figure 1 shows an overview of the location of residences, it was also provided in previous studies^4,5^. Between April 2016 and January 2019, 65 houses and buildings in the evacuation zones of Namie, Futaba, Okuma, and Tomioka towns were surveyed. Of the 65 residences, 51 were made of wood (49 detached houses and 2 community centers), 10 were light steel–framed, and 4 were reinforced concrete (1 detached house and 3 apartment buildings). The houses are one or two stories high, while the apartment buildings are three stories high. Vigorous housecleaning activities were conducted by 2–4 persons in each residence. The cleaning protocol used was as previously described^4^. During cleaning (dusting and vacuuming), all doors and windows were closed, and no ventilation fan was used. Therefore, this cleaning protocol was different from the daily vacuuming routine of the residents. In this research campaign, 271 three-layer non-woven masks and 13 three-dimensional N95 respirators (MK-910-N95DS2V; Kawanishi Industry Co. Ltd., Aichi, Japan) were worn by total of six persons in 58 residences. The particle collection efficiency of the N95 respirators was > 95%, while that of the non-woven masks, which were household masks, was not clearly defined^11^. Several non-woven masks were worn stacked in pairs. For all mask samples, the IDs of the person and residence where the mask was worn and the date when the mask was worn were recorded. The duration of mask-wearing was not recorded for most masks. The working time was approximately 1.5–2.5 h between 9:00 and 17:00 (JST). The ID numbers of the residences and persons were identical to those included in previous studies^5,9^. The worn masks were individually placed in zipper bags to avoid cross-contamination and transferred to the Isotope Science Center of the University of Tokyo for analysis. The masks were labeled “mask-XXX”, based on the date they were worn.

### Radioactivity measurement of the masks

Each mask or N95 respirator stored in a zipper bag was exposed to an imaging plate (IP) (BAS IP MS 3543 E; FUJIFILM Corporation, Tokyo, Japan) for 24 h to estimate the radioactivity of radiocesium in the masks. The total radioactivity of radiocesium in the masks was measured using a previously described method^9^. Each non-woven fabric mask was packed into a cylindrical plastic vessel (47 mm diameter, 6 cm height) to a height of 1.7 cm height, while each N95 respirator was packed to a height of 5.0 cm. The masks were analyzed by a high-resolution gamma spectrometry system with a high-purity germanium detector (GX4018; Mirion Technologies Inc., California, USA). The counting time for each sample was 50,000 s. Radioactivity standard solutions of ^134^Cs (CZ-010; Japan Radioisotope Association) and ^137^Cs (CS-005; Japan Radioisotope Association) were added to unused masks for quantitative measurement. The radioactivity of the standard solutions of both ^134^Cs and ^137^Cs, as calibrated by the Japan Calibration Service System, was 236 Bq as of March 11, 2011. The detection limit of ^134^Cs and ^137^Cs on the masks was 0.070 Bq, each with a counting time of 50,000 s.

### Isolation and radioactivity measurements of cesium-bearing particles

The method for isolating CsMPs from the masks was modified from a previous study^12^. A mask with significant radioactivity was fixed on an IP for 30 min to locate the CsMPs on the mask. IP images were processed using ImageJ software with the ISAC plugin. If a high-radioactivity spot was found in a mask, an approximately 3–5 mm^2^ area around the spot was cut off; then, the fragment was placed in a 3 mL plastic tube. Afterwards, methanol (1 mL) was added to the tube. The tube was sonicated in an ultrasonic washer bath at room temperature for 10 min to transfer the CsMPs from the non-woven fabric to the solution. After the ultrasonic treatment, the non-woven fabric and solution were dropped onto double-sided Kapton tape and air-dried. The Kapton tape was placed on the IP for 30 min to confirm the presence of radioactive spots. Approximately 1 mm^2^ of the Kapton tape around the high radioactivity spots was cut off. Based on previous findings^9,10,12^, high-radioactivity spots indicate the presence of CsMPs. If high-radioactivity spots remained on a fragment of non-woven fabric, the fragment was treated again by ultrasonication. After isolation, the CsMPs were placed on a fragment of Kapton tape at the center of a small plastic case (38 × 66 mm, 13 mm height, and 2 mm bottom thickness). Each isolated CsMP was measured using either of the two high-resolution gamma spectrometry systems with a high-purity germanium detector (NIGC2020; Princeton Gamma-Tech Instruments Inc., New Jersey, USA and GX4018; Mirion Technologies Inc., California, USA) for 200,000–600,000 s. The relative efficiency for 1,333 keV of each germanium crystal was 25.2% for NIGC2020 and 46.5% for GX4018. A CsMP named “Mask-12-A,” found in a mask in a previous study^9^, was used as the radioactivity standard for CsMPs. As of December 2, 2016, the radioactivity of ^134^Cs was 4.68 ± 0.11, while that of ^137^Cs was 25.3 ± 0.1. The detection limits of ^134^Cs and ^137^Cs in CsMPs were 0.00577 Bq and 0.00537 Bq, respectively, with a counting time of 600,000 s. IDs were assigned to the isolated CsMPs in the same way as the mask IDs, but were capitalized; when two or more particles were found in one mask, the ID was followed by a letter.

### Estimation of the diameter of cesium-bearing particles

Assuming that all the CsMPs were spherical and generated in Unit 2, a correlation between ^137^Cs radioactivity (Bq) of type A CsMPs and their particle volume (cm^3^) (*V*) was suggested as the Eq. (1) in a previous study^13^.

1 $$\mathrm{Type \; A \; radioactivity \; of } \; {}^{137}\mathrm{Cs\,}[\mathrm{Bq}] = 2\times {10}^{16}\times {V}^{1.40}$$

Therefore, the diameter of type A CsMPs can be estimated from their radioactivity using the Eq. (2).

2 $$\mathrm{Diameter \; of \; CsMP\,}\left[\mathrm{\upmu m}\right]=2\times {\left(\frac{3\times V\times \pi }{4}\right)}^\frac{1}{3}\times {10}^{4}$$

### Ethical approval

This study was approved by Ethics Committee Tohoku University Graduate School of Pharmaceutical Sciences. All experiments were performed in accordance with relevant guidelines and regulations. For the residents whose houses were examined in this study, the subject, object, sampling and measurement methods, and publication methods were explained verbally or in writing, and written informed consent was obtained from each resident and person who had done housecleaning activities before the examination.

## Results

Table 1 shows the radioactivity of the isolated CsMPs according to the dates of mask-wearing and radioactivity measurement and live time of measurement. Mask-001-A, Mask-012-A, Mask-012-B, and Mask-012-C were reported in the study by Higaki et al.^9^. In this study, 44 new CsMPs were discovered. Therefore, the total number of CsMPs detected during the campaign was 48. The 44 new particles did not exceed 25 Bq; thus, they were not highly radioactive. Such a characteristic of the newly discovered particles is similar to that of the previously reported insoluble CsMPs that originated from Unit 2. It was assumed that they were stably preserved, because the CsMPs released during the accident were present indoors. Okumura et al*.*^14^ reported that CsMPs are dissolved by weathering in the environment rather than by the physical decay of radiocesium. Therefore, the CsMPs were stable under indoor conditions and were unaffected by weathering. CsMPs were not found in the masks worn by Person 5.

**Table 1:** ID of CsMPs detected on the masks worn in this study and in a previous study (Higaki et al. 2017^9^), the ID of the residence, measurement date, live time, and radioactivity of CsMPs.

Sample ID	Residence ID	Measurement date	Live time [s]	^134^Cs [Bq]	σ	^137^Cs [Bq]	σ
Mask-001-A*	ID_09	2016/12/6	200,000	0.193	0.012	1.01	0.01
Mask-012-A*	ID_14	2016/12/2	50,000	4.84	0.116	25.4	0.1
Mask-012-B*	ID_14	2016/12/3	30,000	11.0	0.228	58.8	0.2
Mask-012-C*	ID_14	2016/12/3	30,000	8.62	0.20	46.3	0.2
Mask-001-B	ID_09	2019/7/14	200,000	0.0227	0.0129	0.0973	0.0050
Mask-015	ID_14	2019/8/23	500,000	0.0763	0.0134	0.322	0.005
Mask-023	ID_03	2020/1/22	500,000	0.0572	0.0165	0.345	0.006
Mask-037-A	ID_18	2019/10/1	500,000	0.387	0.031	2.08	0.012
Mask-037-B	ID_18	2019/6/25	500,000	0.185	0.020	1.12	0.009
Mask-045	ID_19	2019/9/17	280,000	0.159	0.025	0.704	0.010
Mask-055	ID_20	2019/10/7	600,000	0.0147	0.0091	0.126	0.004
Mask-058	ID_21	2020/2/27	600,000	0.133	0.017	0.779	0.006
Mask-067	ID_22	2019/7/4	200,000	0.0397	0.0190	0.276	0.009
Mask-083	ID_24	2019/12/20	600,000	0.0763	0.0136	0.464	0.006
Mask-087-A	ID_24	2020/3/10	600,000	0.0260	0.0095	0.146	0.004
Mask-087-B	ID_24	2019/7/23	200,000	0.0752	0.0205	0.547	0.010
Mask-087-C	ID_24	2020/6/17	600,000	LDL	–	0.0468	0.0020
Mask-087-D	ID_24	2020/5/9	515,000	0.0299	0.0121	0.193	0.004
Mask-087-E	ID_24	2020/5/15	600,000	0.0252	0.0095	0.119	0.004
Mask-087-F	ID_24	2020/1/7	600,000	0.0326	0.0102	0.204	0.004
Mask-087-G	ID_24	2020/6/23	600,000	LDL	–	0.0805	0.0023
Mask-090	ID_24	2020/4/15	600,000	0.107	0.017	0.652	0.007
Mask-101	ID_26	2020/4/8	600,000	0.0439	0.0133	0.352	0.005
Mask-114	ID_28	2020/6/10	600,000	0.0241	0.0074	0.132	0.003
Mask-126-A	ID_01	2019/6/17	200,000	0.0965	0.0183	0.571	0.009
Mask-126-B	ID_01	2019/6/19	500,000	0.0556	0.0109	0.353	0.005
Mask-126-C	ID_01	2020/6/5	400,000	0.0513	0.0131	0.363	0.005
Mask-126-D	ID_01	2020/5/29	595,000	0.0334	0.0119	0.261	0.005
Mask-126-E	ID_01	2020/5/22	600,000	0.306	0.028	1.89	0.01
Mask-127	ID_01	2019/8/19	300,000	0.116	0.019	0.681	0.009
Mask-142	ID_30	2019/10/21	350,000	0.0556	0.0155	0.465	0.007
Mask-197	ID_39	2020/1/15	600,000	0.0576	0.0104	0.298	0.005
Mask-242-A	ID_48	2019/4/27	200,000	0.177	0.021	1.71	0.01
Mask-242-B	ID_48	2019/5/9	200,000	0.0699	0.0151	0.633	0.010
Mask-243-A	ID_48	2019/5/16	400,000	0.0971	0.0121	0.850	0.008
Mask-243-B	ID_48	2019/5/22	500,000	0.0343	0.0074	0.352	0.005
Mask-243-C	ID_48	2019/5/17	200,000	0.0552	0.0133	0.441	0.009
Mask-246	ID_49	2019/9/9	500,000	0.0184	0.0067	0.193	0.004
Mask-253	ID_50	2019/10/15	520,000	0.0421	0.0082	0.384	0.006
Mask-256	ID_50	2019/5/24	200,000	0.0645	0.0132	0.661	0.010
Mask-275	ID_56	2019/10/30	500,000	0.0245	0.0070	0.246	0.005
Mask-276	ID_56	2020/4/22	600,000	0.0164	0.0057	0.132	0.004
Mask-282	ID_60	2020/3/17	590,000	0.0377	0.0082	0.421	0.005
Mask-283	ID_61	2020/3/24	600,000	0.0570	0.0098	0.649	0.006
Mask-284-A	ID_61	2019/8/5	200,000	0.0592	0.0111	0.636	0.009
Mask-284-B	ID_61	2020/4/30	600,000	0.0101	0.0059	0.168	0.004
Mask-284-C	ID_61	2019/8/9	600,000	0.0253	0.0052	0.246	0.004
Mask-284-D	ID_61	2020/7/7	600,000	LDL	–	0.0142	0.0015

Radioactivity is decay corrected to date of the mask was worn. Asterisk indicates the CsMPs detected in a previous study (Higaki et al. 2017)^9^.

*LDL*: lower the detection limit.

Supplementary Table S1 online shows the IDs of persons and the residences where the masks were worn, the date and time when the masks were worn, and ^134^Cs and ^137^Cs radioactivity for all mask samples. Among them, mask-001–014 was previously reported^9^. Of the 284 masks worn in this study, significant ^137^Cs radioactivity was detected in 268.

Supplementary Table S2 online shows the percentage of CsMPs found in each class based on the ^137^Cs radioactivity in the masks. These CsMPs were occasionally detected when the ^137^Cs radioactivity of the mask was greater than 10 Bq. The higher the ^137^Cs radioactivity of the mask, the higher the frequency of the CsMPs.

Table 2 shows the estimated volume (cm^3^) and diameter (μm) of each CsMP calculated from the ^137^Cs radioactivity decay-corrected on March 11, 2011. CsMPs with diameters less than 4.30 μm were trapped by the masks, while CsMPs with diameters greater than 1.33 μm were isolated from the masks. Based on its ^137^Cs radioactivity, the diameter of Mask-001-A was estimated to be 3.61 μm. However, the size of an SEM image was 2.1 μm^9^, and this size was the only estimate.

**Table 2:** Estimated particle size of CsMPs based on its radioactivity.

Sample-ID	^134^Cs [Bq]	σ	^137^Cs [Bq]	σ	Estimated volume [cm^−3^]	Estimated diameter [μm]
Mask-001-A*	1.25	0.08	1.15	0.01	2.51 × 10^–12^	3.61
Mask-001-B	0.147	0.083	0.111	0.006	4.72 × 10^–13^	2.07
Mask-015	0.506	0.089	0.367	0.006	1.11 × 10^–12^	2.76
Mask-023	0.379	0.109	0.393	0.006	1.17 × 10^–12^	2.80
Mask-037-A	2.76	0.22	2.38	0.01	4.22 × 10^–12^	4.30
Mask-037-B	1.32	0.15	1.28	0.01	2.71 × 10^–12^	3.71
Mask-045	1.14	0.18	0.806	0.012	1.95 × 10^–12^	3.32
Mask-053	0.106	0.066	0.144	0.004	5.70 × 10^–13^	2.21
Mask-058	0.956	0.125	0.893	0.007	2.09 × 10^–12^	3.40
Mask-067	0.309	0.148	0.318	0.010	1.00 × 10^–12^	2.66
Mask-083	0.608	0.108	0.535	0.007	1.45 × 10^–12^	3.01
Mask-087-A	0.208	0.076	0.169	0.004	6.37 × 10^–13^	2.29
Mask-087-B	0.600	0.164	0.631	0.011	1.64 × 10^–12^	3.14
Mask-087-C	LDL	–	0.0540	0.0023	2.82 × 10^–13^	1.75
Mask-087-D	0.239	0.096	0.223	0.005	7.78 × 10^–13^	2.45
Mask-087-E	0.201	0.076	0.138	0.004	5.51 × 10^–13^	2.18
Mask-087-F	0.260	0.081	0.235	0.005	8.08 × 10^–13^	2.48
Mask-087-G	LDL	–	0.0928	0.0026	4.16 × 10^–13^	1.99
Mask-090	0.853	0.139	0.752	0.008	1.85 × 10^–12^	3.27
Mask-101	0.353	0.107	0.406	0.006	1.19 × 10^–12^	2.82
Mask-114	0.198	0.061	0.153	0.003	5.94 × 10^–13^	2.24
Mask-126	0.976	0.156	0.788	0.011	1.92 × 10^–12^	3.31
Mask-127-A	0.810	0.154	0.661	0.011	1.69 × 10^–12^	3.17
Mask-127-B	0.467	0.092	0.408	0.006	1.20 × 10^–12^	2.83
Mask-127-C	0.431	0.110	0.420	0.006	1.22 × 10^–12^	2.85
Mask-127-D	0.280	0.100	0.302	0.005	9.65 × 10^–13^	2.63
Mask-127-E	2.57	0.24	2.19	0.01	3.98 × 10^–12^	4.22
Mask-142	0.489	0.136	0.540	0.009	1.46 × 10^–12^	3.02
Mask-197	0.540	0.097	0.347	0.006	1.07 × 10^–12^	2.72
Mask-242-A	2.00	0.24	2.02	0.02	3.75 × 10^–12^	4.13
Mask-242-B	0.790	0.171	0.748	0.012	1.85 × 10^–12^	3.26
Mask-243-A	1.10	0.14	1.00	0.01	2.28 × 10^–12^	3.50
Mask-243-B	0.388	0.084	0.416	0.006	1.21 × 10^–12^	2.84
Mask-243-C	0.624	0.150	0.521	0.011	1.43 × 10^–12^	2.99
Mask-246	0.216	0.079	0.228	0.005	7.91 × 10^–13^	2.46
Mask-253	0.531	0.103	0.457	0.007	1.30 × 10^–12^	2.90
Mask-256	0.813	0.166	0.787	0.012	1.91 × 10^–12^	3.30
Mask-275	0.328	0.093	0.294	0.006	9.48 × 10^–13^	2.61
Mask-276	0.220	0.077	0.158	0.004	6.08 × 10^–13^	2.25
Mask-282	0.531	0.116	0.505	0.007	1.40 × 10^–12^	2.97
Mask-283	0.803	0.139	0.779	0.008	1.90 × 10^–12^	3.30
Mask-284-A	0.833	0.156	0.763	0.010	1.87 × 10^–12^	3.28
Mask-284-B	0.142	0.083	0.202	0.005	7.24 × 10^–13^	2.39
Mask-284-C	0.356	0.073	0.296	0.005	9.51 × 10^–12^	2.62
Mask-284-D	LDL	–	0.0170	0.0018	1.24 × 10^–13^	1.33

*LDL*: Lower the detection limit.

Asterisk indicates CsMPs detected in a previous study (Higaki et al. 2017)^9^.

## Discussion

**Figure 2:**
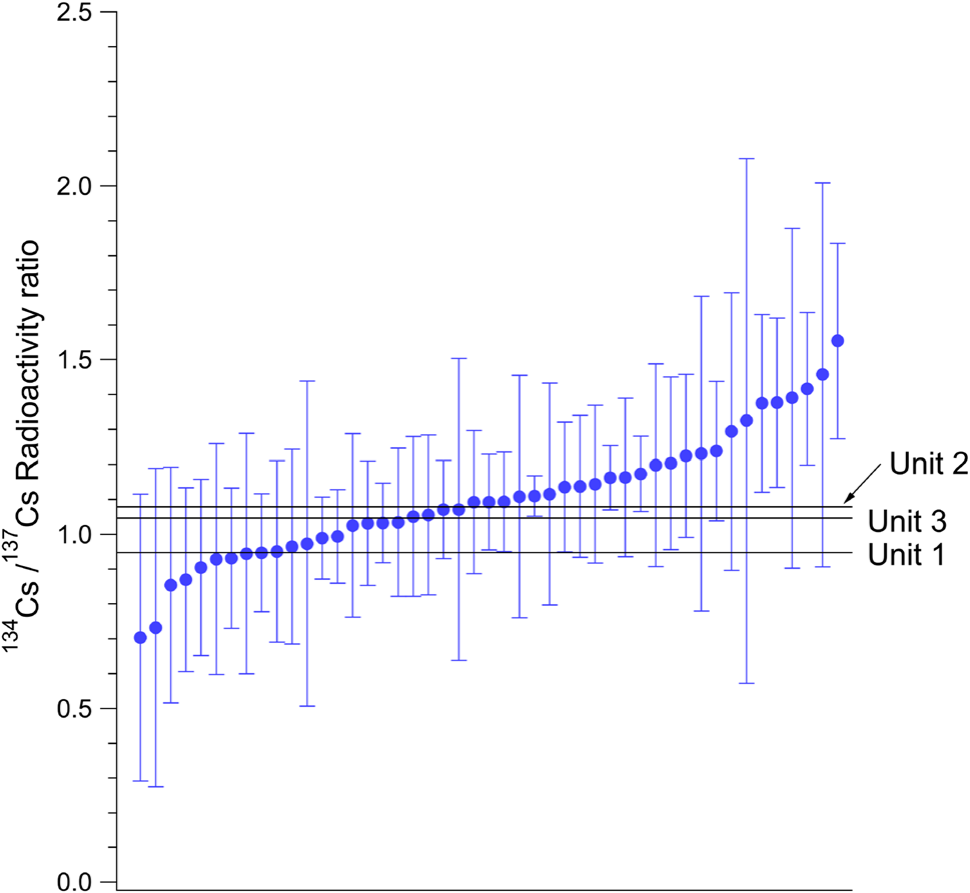
Radioactivity ratio of ^134^Cs/^137^Cs in the CsMPs detected in this study and that of Higaki et al. 2017^9^ corrected for decay on March 11, 2011 and arranged in decreasing order.

Figure 2 shows the radioactivity ratios—corrected for decay on March 11, 2011—of ^134^Cs/^137^Cs in the CsMPs detected in this study and that of Higaki et al*.*^9^, in decreasing order. According to Nishihara et al*.*^15^, the radioactivity ratios calculated from the cumulative hours of nuclear fuel were 0.941, 1.08, and 1.05 for Units 1, 2, and 3, respectively. However, measuring the radioactivity of CsMPs after 9 years since the accident yielded large measurement errors due to the extremely small count per second of ^134^Cs, as a result of ^134^Cs decay. Therefore, it was difficult to determine the origin of the CsMPs based solely on the ratio of most particles. However, except for the amorphous particles reported by Higaki et al*.*^9^, all particles with radioactivity of a few Bq of ^137^Cs were Type A particles^13^.

Several non-woven masks were worn stacked in pairs. The non-woven masks worn in this study did not have a clearly defined particle collection efficiency. In addition, some leakage between the mask and face was considered. The collection efficiency of radiocesium by mask was determined using the following Eq. (3).

3 $$\mathrm{collection \; efficiency \; of \; radiocesium \; by \; mask} \; \left[\%\right]=\left(1-\frac{radioactivity \; of \; {}^{137}Cs \; in \; inner \; mask}{radioactivity\; of \; {}^{137}Cs \; in \; outer \; mask}\right)\times 100$$

Table 3 lists the collection efficiency of the masks. Significant collection efficiency was determined in 51 of 76 cases; the rest of the cases had collection efficiencies of less than 0. The maximum, minimum, and average collection efficiencies were 81.7%, 1.4%, and 34.7%, respectively. Here, these collection efficiencies might be overestimated because air flow through the inner mask was cleaned from coarser particles by the outer mask. Therefore, these actual efficiencies were equal to or less than the obtained values. In addition, CsMPs were trapped by four outer and eight inner masks. This finding is attributed to the performance limitations of the masks worn in this campaign because no individual differences in collection efficiency were observed.

**Table 3:** Ratio of radioactivity in the inner mask to that in the outer mask.

Mask ID	Residence ID	Type	Person No.	^137^Cs [Bq]	Estimated collection efficiency (%)
Mask-032	ID_18	Outer	1	0.785	56.6
Mask-034	ID_18	Outer	3	6.07	78.7
Mask-038	ID_18	Outer	3	14.4	15.4
Mask-040	ID_19	Outer	1	2.72	4.96
Mask-042	ID_19	Outer	3	6.47	67.9
Mask-050	ID_20	Outer	2	87.7	30.9
Mask-052	ID_20	Outer	3	8.35	55.7
Mask-054	ID_20	Outer	1	102	31.1
Mask-056	ID_20	Outer	3	7.91	8.73
Mask-058	ID_21	Outer	1	92.1	34.3
Mask-060	ID_21	Outer	2	130	34.3
Mask-062	ID_21	Outer	3	20.5	55.5
Mask-064	ID_22	Outer	1	50.7	42.1
Mask-068	ID_23	Outer	1	38.4	56.5
Mask-073	ID_23	Outer	3	13.4	56.5
Mask-074	ID_24	Outer	3	4.26	36.7
Mask-076	ID_24	Outer	4	9.05	24.1
Mask-078	ID_24	Outer	1	39.7	21.0
Mask-080	ID_24	Outer	3	23.9	18.7
Mask-082	ID_24	Outer	4	32.7	35.0
Mask-084	ID_24	Outer	1	57.9	37.4
Mask-090	ID_24	Outer	4	96.4	1.37
Mask-092	ID_24	Outer	4	75.8	2.53
Mask-094	ID_25	Outer	1	161	44.4
Mask-096	ID_25	Outer	3	2.75	66.4
Mask-098	ID_25	Outer	4	2.42	25.3
Mask-100	ID_26	Outer	1	13.8	24.0
Mask-102	ID_26	Outer	2	23.9	24.5
Mask-104	ID_26	Outer	3	14.5	5.04
Mask-106	ID_26	Outer	5	9.95	9.23
Mask-110	ID_27	Outer	3	3.56	40.3
Mask-112	ID_27	Outer	5	7.87	22.0
Mask-122	ID_01, 02	Outer	5	108	5.25
Mask-131	ID_29	Outer	5	21.6	38.9
Mask-133	ID_29	Outer	1	21.5	25.8
Mask-139	ID_30	Outer	4	5.48	81.7
Mask-143	ID_30	Outer	2	21.3	12.2
Mask-149	ID_31	Outer	1	6.32	27.5
Mask-155	ID_32	Outer	3	1.02	23.8
Mask-157	ID_32	Outer	4	0.534	33.6
Mask-163	ID_32	Outer	4	0.667	33.9
Mask-166	ID_33	Outer	1	15.0	5.31
Mask-168	ID_34	Outer	1	3.96	45.1
Mask-171	ID_35	Outer	1	55.4	75.6
Mask-181	ID_33,34,35	Outer	5	2.43	51.5
Mask-183	ID_33,34,35	Outer	3	1.67	33.2
Mask-187	ID_36	Outer	5	3.11	46.6
Mask-190	ID_37	Outer	5	1.12	48.0
Mask-201	ID_40A	Outer	2	8.53	16.3
Mask-254	ID_50	Outer	1	5.75	19.1
Mask-263	ID_51	Outer	1	10.9	79.4

**Figure 3:**
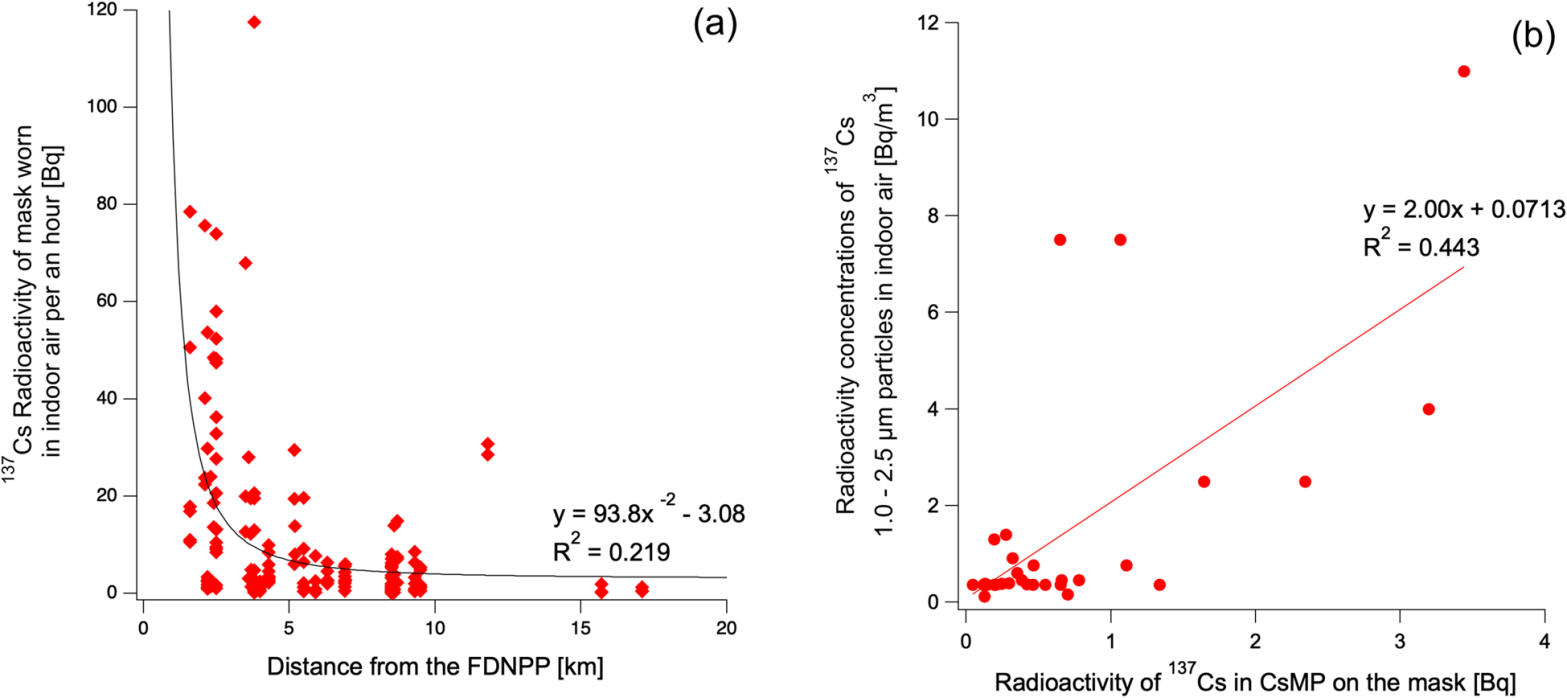
(**a**) Relationship between the distance of the residences from the FDNPP and the total radioactivity of the masks. (**b**) Relationship between radioactivity of ^137^Cs in CsMP in the mask (excluding mask-012, where non-spherical CsMPs were found) and the radioactivity of 1–2.5 µm particles in indoor air reported by Yoshida-Ohuchi and Shinohara (2020). Radioactivity is decay corrected to March 11, 2011.

The correlation between indoor pollution and the concentration of radiocesium trapped by the masks was investigated. Figure 3a shows an inverse square relationship between the distance of the residences from the FDNPP and the concentration of ^137^Cs radioactivity in each mask; such a relationship was earlier reported by Yoshida-Ohuchi and Shinohara^5^. A moderate association (R^2^ = 0.219) was also observed. This reflects the fact that house dust that was scattered indoors owing to cleaning activities was collected by masks, regardless of the person cleaning. The same study by Yoshida-Ohuchi and Shinohara^5^ also showed the concentration of ^137^Cs radioactivity for each house dust particle size (< 0.25 µm, 0.25–0.5 µm, 0.5–1.0 µm, 1.0–2.5 µm, 2.5–6.6 µm, > 6.6 µm, total, PM2.5) in each residence. A Type A CsMP, which is a typical size, has a size of 1.0–2.5 µm. Figure 3b shows the relationship between the radioactivity of CsMPs in the masks (excluding Mask-012, where non-spherical CsMPs were found) and the radioactivity of particles with a size of 1.0–2.5 µm. The correlation coefficient was R^2^ = 0.444, which is statistically significant. The relationships among the other particle sizes are shown in Supplementary Fig. S1 online. Thus, the R^2^ values below 0.1 for the other particle sizes indicate no correlation. This finding indicates that CsMPs had a large proportion of radioactivity for particles in the size range of 1.0–2.5 µm concerning the radiocesium radioactivity of particles for indoor air contamination.

Table 4 presents the ^137^Cs radioactivity ratios of the CsMPs to the total ^137^Cs radioactivity of the masks. The maximum ratio was approximately 4%, excluding N95 respirators. The N95 respirators accounted for a slightly higher percentage of ^137^Cs radioactivity. Such a finding is attributed to the ability of N95 respirators to adsorb dust containing CsMPs; thus, larger particles were repelled rather than adsorbed by the masks. The contamination of the residences near the FDNPP reflects the accumulation of multiple plumes released from the FDNPP. Nineteen plumes with concentrations below 100 Bq/m^3^ were released between March 12 and March 25, 2011^16^. Of these, 11 plumes passed through the northern Hamadori area, while 9 plumes passed through the southern Hamadori area (one plume passed through both). However, only few of these releases contained CsMPs. CsMPs released from Unit 2 were detected in two plumes on March 15 but not in the plumes after March 20^17^. Therefore, the radioactivity of CsMPs relative to the total radiocesium in indoor contamination may be low. Soluble radiocesium may remain indoors in its original form without weathering or soluble radioactive cesium may be present in house dust, as evidenced by the possibility of soluble cesium that may have dissolved during the separation process.

**Table 4:** Ratio of CsMP radioactivity to the total radioactivity of a mask containing CsMPs.

Sample ID	Worn	Radioactivity of CsMP at the worn				Radioactivity of mask				^137^Cs radioactivity ratio of CsMP/Mask(%)
		^134^Cs [Bq]	σ	^137^Cs [Bq]	σ	^134^Cs [Bq]	σ	^137^Cs [Bq]	σ	
Mask-001-A	2016/9/29	0.193	0.012	1.01	0.01	7.25	0.10	39.0	0.3	2.59
Mask-001-B	2016/9/29	0.0227	0.0128	0.0973	0.0050	7.25	0.10	39.0	0.3	0.250
Mask-012-A	2016/10/25	4.84	0.12	25.4	0.1	238	1	1420	3	1.79
Mask-012-B	2016/10/25	11.0	0.2	58.8	0.2	238	1	1420	3	4.14
Mask-012-C	2016/10/25	8.62	0.20	46.3	0.2	238	1	1420	3	3.26
Mask-015	2016/10/26	0.0763	0.0134	0.322	0.005	33.4	0.2	195	1	0.165
Mask-023	2016/10/26	0.0571	0.0165	0.345	0.006	10.1	0.1	57.2	0.4	0.603
Mask-037-A	2017/1/17	0.386	0.030	2.08	0.01	10.0	0.2	61.2	0.5	3.40
Mask-037-B	2017/1/17	0.185	0.020	1.12	0.01	10.0	0.2	61.2	0.5	1.82
Mask-045	2017/1/18	0.159	0.025	0.704	0.010	7.73	0.15	47.8	0.5	1.47
Mask-053	2017/1/24	0.0147	0.0091	0.126	0.004	11.7	0.2	70.1	0.6	0.180
Mask-058	2017/1/25	0.133	0.017	0.779	0.006	15.1	0.2	92.1	0.7	0.846
Mask-067	2017/4/20	0.0397	0.0189	0.276	0.009	4.63	0.12	30.7	0.4	0.900
Mask-083	2017/5/16	0.0762	0.0136	0.464	0.006	3.20	0.10	21.3	0.3	2.18
Mask-087-A	2017/5/17	0.0260	0.0095	0.146	0.004	9.25	0.16	65.9	0.6	0.222
Mask-087-B	2017/5/17	0.0751	0.0205	0.547	0.010	9.25	0.16	65.9	0.6	0.831
Mask-087-C	2017/5/17	LDL	–	0.0468	0.0020	9.25	0.16	65.9	0.6	0.071
Mask-087-D	2017/5/17	0.0299	0.0121	0.193	0.004	9.25	0.16	65.9	0.6	0.294
Mask-087-E	2017/5/17	0.0251	0.0095	0.119	0.004	9.25	0.16	65.9	0.6	0.181
Mask-087-F	2017/5/17	0.0326	0.0102	0.204	0.004	9.25	0.16	65.9	0.6	0.309
Mask-087-G	2017/5/17	LDL	–	0.0805	0.0023	9.25	0.16	65.9	0.6	0.122
Mask-090	2017/5/17	0.107	0.017	0.652	0.007	14.1	1.9	96.4	0.7	0.676
Mask-101	2017/5/25	0.0439	0.0133	0.352	0.005	1.55	0.08	10.5	0.2	3.35
Mask-114	2017/6/15	0.0248	0.0076	0.130	0.003	1.65	0.09	12.2	0.2	1.06
Mask-126	2017/7/11	0.116	0.019	0.681	0.009	18.8	0.2	131	1	0.520
Mask-127-A	2017/7/11	0.0964	0.0183	0.571	0.009	12.2	0.2	84.4	0.6	0.677
Mask-127-B	2017/7/11	0.0556	0.0109	0.353	0.005	12.2	0.2	84.4	0.6	0.418
Mask-127-C	2017/7/11	0.0529	0.0135	0.357	0.005	12.2	0.2	84.4	0.6	0.423
Mask-127-D	2017/7/11	0.0334	0.0119	0.261	0.005	12.2	0.2	84.4	0.6	0.309
Mask-127-E	2017/7/11	0.305	0.028	1.89	0.01	12.2	0.2	84.4	0.6	2.24
Mask-142	2017/8/29	0.0556	0.0155	0.465	0.007	3.08	0.11	24.2	0.3	1.92
Mask-197	2017/11/8	0.0575	0.0104	0.298	0.005	2.11	0.09	16.6	0.3	1.79
Mask-242-A	2018/5/30	0.177	0.021	1.71	0.01	1.37	0.05	12.9	0.2	13.2
Mask-242-B	2018/5/30	0.0699	0.0151	0.633	0.010	1.37	0.05	12.9	0.2	4.90
Mask-243-A	2018/5/30	0.0970	0.0121	0.850	0.008	4.15	0.07	39.3	0.3	2.16
Mask-243-B	2018/5/30	0.0343	0.0074	0.352	0.005	4.15	0.07	39.3	0.3	0.897
Mask-243-C	2018/5/30	0.0552	0.0133	0.441	0.009	4.15	0.07	39.3	0.3	1.12
Mask-246	2018/7/9	0.0184	0.0067	0.193	0.004	0.871	0.072	8.34	0.21	2.31
Mask-253	2018/9/26	0.0421	0.0082	0.384	0.006	1.03	0.07	8.87	0.21	4.32
Mask-256	2018/9/26	0.0645	0.0132	0.661	0.010	0.880	0.036	8.69	0.13	7.60
Mask-275	2018/11/30	0.0245	0.0070	0.246	0.005	1.30	0.11	14.1	0.3	1.75
Mask-276	2018/11/30	0.0164	0.0057	0.132	0.004	2.18	0.05	24.3	0.2	0.543
Mask-282	2019/1/23	0.0377	0.0082	0.421	0.005	5.72	0.18	60.2	0.5	0.700
Mask-283	2019/1/24	0.0570	0.0098	0.649	0.006	3.41	0.19	34.4	0.4	1.89
Mask-284-A	2019/1/24	0.0591	0.0111	0.636	0.009	17.9	0.3	196	1	0.324
Mask-284-B	2019/1/24	0.0101	0.0059	0.168	0.004	17.9	0.3	196	1	0.0859
Mask-284-C	2019/1/24	0.0253	0.0052	0.246	0.004	17.9	0.3	196	1	0.126
Mask-284-D	2019/1/24	LDL	–	0.0142	0.0015	17.9	0.3	196	1	0.00723

*LDL*: Lower the detection limit.

**Figure 4:**
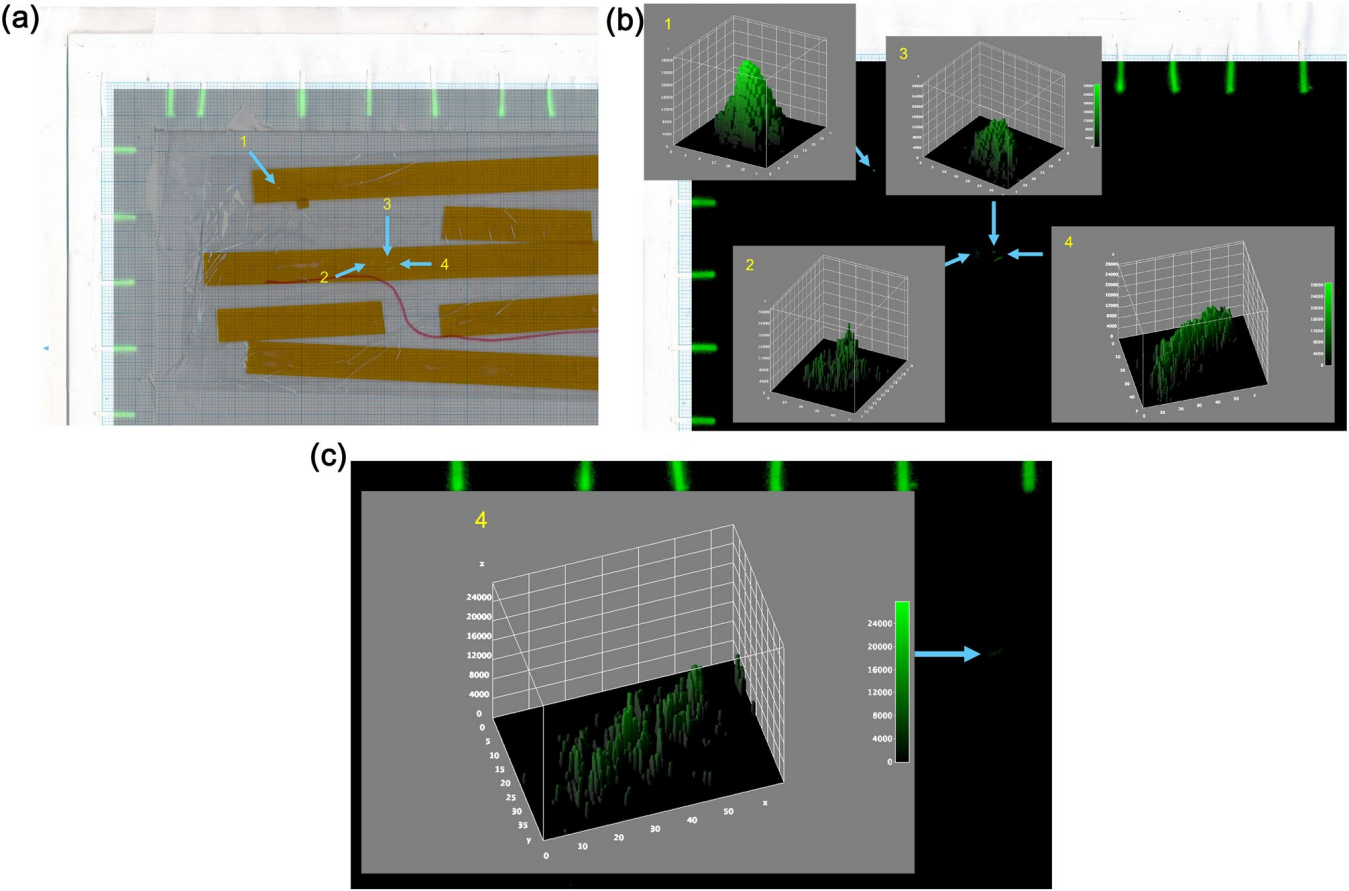
(**a**) A fusion image of IP image and Kapton tape during the isolation of CsMPs from masks. Point 1 was from mask-284, points 2–4 were from mask-282. (**b**) 3D surface plot of the IP image around the location of count accumulation with high contrast. Point 1 shows typical Type A CsMP distribution, and points 2–4 show a broad asymmetric distribution. The unit for the X and Y axis of the 3D surface plot are pixels of two-dimensional distribution, the Z axis is cumulative counts of photo-stimulated luminescence by 30 min exposure. (**c**) 3D surface plot of the IP image around the location of count same Kapton tape fragment (point 4) after it had been sonicated with ethanol with accumulation with high contrast.

Kapton tape was placed on an imaging plate for 30 min to confirm the presence of radioactive spots. A 30-min exposure only visualizes the points where radiocesium accumulates at high concentrations. Figure 4a shows the CsMPs during the isolation process. A high bar count indicates the filter paper containing ^137^Cs solution used for alignment. Figure 4b indicates a 3D surface plot of the area using ImageJ with a high contrast. If the CsMPs were spherical, the count distribution would be circular with the highest count at the center. The upper point (point 1) shows this distribution. However, the bottom three points (2, 3, and 4), which exhibit a broad asymmetric distribution, are not shown. The lower-right point was cut from the Kapton tape, sonicated with ethanol, and IP imaged for 30 min (Fig. 4c). The 3D surface plot shows a similar planar distribution but with lower counts on the vertical axis. Subsequently, the Kapton tape was sonicated with ethanol, and IP images were captured for 30 min. No significant IP images were obtained. This is presumably due to the presence of high concentrations of soluble radiocesium particles or aerosols adhered to house dust. It is presumed that the soluble material gradually dissolved, thereby lowering the concentration of radiocesium. Alternatively, it is also possible that the CsMPs were irregularly shaped and easily dissolved, or their structure became brittle over time. CsMPs are primarily destroyed through outdoor weathering^14,18^. However, even indoors, the radiation emitted from radioactive cesium may gradually damage the chemical bonds of the main structure (SiO_2_) and accumulate over 8 years since the accident, which could have resulted in the destruction of the CsMPs. The presence of these particles should be examined in detail in future studies.

It should be noted that the cleaning protocol used in this study was different from the vacuum cleaning commonly done by residents, which includes opening all doors and windows and operating a ventilation fan. According to a previous study^4^, vacuum cleaning rarely causes the resuspension of house dust. Therefore, our results show the maximum value considering the assumptions made. When evacuees return to their residences to clean, the actual radioactivity of particles adhered on masks will be much lower, as the returnees can ensure adequate ventilation during cleaning activities. Regardless, radiation exposure should be kept to a minimum and wearing a mask can reduce inhalation exposure. However, the collection efficiency of the masks used in this experiment was only 35% at maximum. Therefore, when cleaning houses around the FDNPP, it is necessary to wear a mask with adequate particle filters and keep the mask in place at all times during cleaning.

## Conclusion

Following previous research, we measured the radioactivity of radiocesium and CsMPs that adhered to the non-woven masks used during the indoor cleaning of 59 residences near the FDNPP. We discovered 44 new CsMPs in 28 of the 284 masks worn. Moreover, we found that CsMPs constituted a large proportion of the radioactivity of particles in the size range of 1.0–2.5 µm in the indoor air. During the isolation process of CsMP, we obtained results that suggest highly concentrated and soluble radiocesium particles or aerosols can adhere to house dust. Some particles were highly radioactive. Therefore, it is desirable to wear masks during cleaning to ensure proper fit. The results obtained in this study suggest that high concentrations of soluble radiocesium particles or aerosols were found adhering to house dust. However, due to the limited number of samples, it was not possible to investigate their characteristics in detail. Additionally, it is possible that the structure of CsMP may naturally deteriorate due to the radioactive decay of radiocesium, even in indoor environments that are unaffected by the weather. The presence of high concentrations of soluble radiocesium particles or aerosols, as well as the detailed mechanisms of their decay, will need to be investigated further.

## Supplementary Information

Supplementary Information.

## Data Availability

The datasets generated and/or analyzed during the current study are available from the corresponding author on reasonable request.
