# Supplementary material for: Radiocesium-bearing microparticles discovered on masks worn during indoor cleaning
Source: Sci Rep. 2023 Jun 20;13:10008. doi: 10.1038/s41598-023-37191-0 (PMC10282055; doi:10.1038/s41598-023-37191-0)
Supplement: Supplementary file 1 — Supplementary Information. [file 41598_2023_37191_MOESM1_ESM.pdf]

Supplementary information

Radiocesium-bearing microparticles discovered on masks worn during indoor cleaning

Authors

Shogo Higaki[1]*, Hiroko Yoshida-Ohuchi[2] and Naohide Shinohara[3]

Affiliations

[1] Isotope Science Center, The University of Tokyo, JAPAN

[2] Cyclotron and Radioisotope Center, Tohoku University, JAPAN

[3] National Institute of Advanced Industrial Science and Technology, JAPAN

Supplementary Table S1 ID of the mask, the ID of the residence where it was worn, ID of the person, type of mask, date and time of mask-wearing, and radioactivity concentration. Radioactivity is decay corrected to the date the mask was worn.

Mask ID	Residence ID	Type	Person No.	Worn	Worn Time	^{134}Cs [Bq]	σ	^{137}Cs [Bq]	σ
mask-001	ID_09	Single	1	2016/9/29	2:00	7.25	0.10	39.0	0.3
mask-002	ID_09	Single	2	2016/9/29	2:00	10.8	0.1	59.1	0.0
mask-003	ID_09	Single	1	2016/9/29	2:00	2.12	0.06	12.0	0.2
mask-004	ID_10	Single	1	2016/10/12	3:40	1.85	0.03	10.0	0.1
mask-005	ID_10	Single	3	2016/10/12	4:20	4.76	0.13	26.0	0.4
mask-006	ID_11	Single	1	2016/10/14	3:30	1.26	0.08	7.20	0.20
mask-007	ID_11	Single	3	2016/10/14	3:30	0.550	0.064	2.47	0.12
mask-008	ID_12	Single	1	2016/10/14	2:50	9.68	0.12	55.5	0.4
mask-009	ID_12	Single	3	2016/10/14	2:50	5.98	0.10	34.7	0.3
mask-010	ID_13	Single	3	2016/10/21	4:50	1.94	0.09	10.3	0.2
mask-011	ID_13	Single	1	2016/10/21	2:30	7.70	0.15	46.5	0.5
mask-012	ID_14	Single	1	2016/10/25	3:50	238	1	1420	3
mask-013	ID_14	Single	2	2016/10/25	2:00	7.86	0.15	44.9	0.5
mask-014	ID_14	Single	3	2016/10/25	3:50	26.8	0.3	154	1
mask-015	ID_14	Single	1	2016/10/26	2:35	33.4	0.2	195	1
mask-016	ID_14	Single	3	2016/10/26	2:35	10.6	0.1	61.4	0.4
mask-017	ID_15	Single	1	2016/10/25	2:00	6.91	0.11	40.1	0.3
mask-018	ID_15	Single	2	2016/10/25	2:00	22.5	0.3	136	1
mask-019	ID_15	Single	3	2016/10/25	2:00	4.42	0.13	25.5	0.3
mask-020	ID_16	Single	1	2016/11/2	4:50	39.2	0.2	234	1
mask-021	ID_16	Single	3	2016/11/2	5:10	11.1	0.1	70.4	0.4
mask-022	ID_03	Single	1	2016/10/26	1:20	7.23	0.11	41.0	0.3
mask-023	ID_03	Single	3	2016/10/26	2:00	10.1	0.1	57.2	0.4
mask-024	ID_06	Single	1	2016/6/23	2:00	3.21	0.11	16.9	0.3
mask-025	ID_06	Single	3	2016/6/23	2:00	3.93	0.12	20.0	0.3
mask-026	ID_07	Single	1	2016/7/26	-	15.4	0.1	82.1	0.4
mask-027	ID_07	Single	3	2016/7/26	-	11.8	0.2	62.7	0.5
mask-028	ID_08	Single	1	2016/7/27	-	7.10	0.15	36.6	0.4
mask-029	ID_08	Single	3	2016/7/27	-	3.15	0.10	16.4	0.3
mask-030	ID_17	Single	1	2017/1/11	4:00	0.839	0.019	4.95	0.06
mask-031	ID_17	Single	3	2017/1/11	4:30	2.43	0.10	15.3	0.3
mask-032	ID_18	Outer	1	2017/1/17	-	LDL	-	0.785	0.079
mask-033	ID_18	Inner	1	2017/1/17	-	LDL	-	0.341	0.064
mask-034	ID_18	Outer	3	2017/1/17	-	1.00	0.07	6.07	0.18
mask-035	ID_18	Inner	3	2017/1/17	-	LDL	-	1.29	0.10
mask-036	ID_18	Outer	1	2017/1/17	-	9.31	0.16	56.0	0.5
mask-037	ID_18	Inner	1	2017/1/17	-	10.0	0.2	61.2	0.5
mask-038	ID_18	Outer	3	2017/1/17	-	2.39	0.09	14.4	0.3
mask-039	ID_18	Inner	3	2017/1/17	-	2.03	0.09	12.2	0.3
mask-040	ID_19	Outer	1	2017/1/18	-	0.518	0.058	2.72	0.14
mask-041	ID_19	Inner	1	2017/1/18	-	0.410	0.054	2.58	0.12
mask-042	ID_19	Outer	3	2017/1/18	-	1.03	0.07	6.47	0.19
mask-043	ID_19	Inner	3	2017/1/18	-	0.543	0.070	2.08	0.12
mask-044	ID_19	Outer	1	2017/1/18	-	2.63	0.04	15.4	0.1
mask-045	ID_19	Inner	1	2017/1/18	-	7.73	0.15	47.8	0.5
mask-046	ID_19	Outer	3	2017/1/18	-	0.938	0.073	5.38	0.17
mask-047	ID_19	Inner	3	2017/1/18	-	2.27	0.07	13.5	0.2
mask-048	ID_20	Single	1	2017/1/24	-	LDL	-	1.30	0.10
mask-049	ID_20	Single	2	2017/1/24	-	0.309	0.064	1.99	0.11
mask-050	ID_20	Outer	2	2017/1/24	-	14.0	0.2	87.7	0.6
mask-051	ID_20	Inner	2	2017/1/24	-	9.53	0.35	60.6	0.5
mask-052	ID_20	Outer	3	2017/1/24	-	1.40	0.08	8.35	0.21
mask-053	ID_20	Inner	3	2017/1/24	-	0.644	0.064	3.70	0.14
mask-054	ID_20	Outer	1	2017/1/24	-	16.3	0.2	102	1
mask-055	ID_20	Inner	1	2017/1/24	-	11.7	0.2	70.1	0.6
mask-056	ID_20	Outer	3	2017/1/24	-	1.32	0.07	7.91	0.21
mask-057	ID_20	Inner	3	2017/1/24	-	1.28	0.08	7.22	0.20
mask-058	ID_21	Outer	1	2017/1/25	-	15.1	0.2	92.1	0.7
mask-059	ID_21	Inner	1	2017/1/25	-	9.93	0.17	60.5	0.5

mask-060	ID_21	Outer	2	2017/1/25	-	21.2	0.2	130	1
mask-061	ID_21	Inner	2	2017/1/25	-	14.2	0.2	85.6	0.6
mask-062	ID_21	Outer	3	2017/1/25	-	3.38	0.11	20.5	0.3
mask-063	ID_21	Inner	3	2017/1/25	-	1.41	0.08	9.13	0.22
mask-064	ID_22	Outer	1	2017/4/20	-	7.97	0.15	50.7	0.5
mask-065	ID_22	Inner	1	2017/4/20	-	4.65	0.12	29.3	0.4
mask-066	ID_22	Single	2	2017/4/20	-	22.5	0.2	149	1
mask-067	ID_22	Single	3	2017/4/20	-	4.63	0.12	30.7	0.4
mask-068	ID_23	Outer	1	2017/4/20	-	3.18	0.10	38.4	0.3
mask-069	ID_23	Inner	1	2017/4/20	-	2.58	0.09	16.7	0.3
mask-070	ID_23	Outer	2	2017/4/20	-	3.99	0.11	25.7	0.3
mask-071	ID_23	Inner	2	2017/4/20	-	12.6	0.2	80.1	0.6
mask-072	ID_23	Inner	3	2017/4/20	-	0.824	0.065	5.81	0.18
mask-073	ID_23	Outer	3	2017/4/20	-	2.03	0.09	13.4	0.3
mask-074	ID_24	Outer	3	2017/5/16	2:30	0.667	0.061	4.26	0.15
mask-075	ID_24	Inner	3	2017/5/16	2:30	0.412	0.059	2.70	0.02
mask-076	ID_24	Outer	4	2017/5/16	1:00	1.46	0.08	9.05	0.21
mask-077	ID_24	Inner	4	2017/5/16	-	1.06	0.06	6.87	0.19
mask-078	ID_24	Outer	1	2017/5/16	3:00	5.77	0.13	39.7	0.4
mask-079	ID_24	Inner	1	2017/5/16	3:00	4.66	0.12	31.3	0.4
mask-080	ID_24	Outer	3	2017/5/16	2:30	3.70	0.07	23.9	0.3
mask-081	ID_24	Inner	3	2017/5/16	-	2.85	0.10	19.4	0.3
mask-082	ID_24	Outer	4	2017/5/16	2:30	5.05	0.12	32.7	0.4
mask-083	ID_24	Inner	4	2017/5/16	2:30	3.20	0.10	21.3	0.3
mask-084	ID_24	Outer	1	2017/5/16	1:00	8.68	0.16	57.9	0.5
mask-085	ID_24	Inner	1	2017/5/16	1:00	5.48	0.13	36.3	0.4
mask-086	ID_24	Outer	3	2017/5/17	2:00	5.92	0.13	41.3	0.4
mask-087	ID_24	Inner	3	2017/5/17	2:00	9.25	0.16	65.9	0.6
mask-088	ID_24	Outer	3	2017/5/17	1:00	4.36	0.11	27.7	0.4
mask-089	ID_24	Inner	3	2017/5/17	1:00	7.19	0.14	52.4	0.5
mask-090	ID_24	Outer	4	2017/5/17	2:00	14.1	1.9	96.4	0.7
mask-091	ID_24	Inner	4	2017/5/17	2:00	14.3	0.2	95.1	0.7
mask-092	ID_24	Outer	4	2017/5/17	-	11.2	0.2	75.8	0.6
mask-093	ID_24	Inner	4	2017/5/17	1:00	11.1	0.2	73.9	0.6
mask-094	ID_25	Outer	1	2017/5/17	3:00	22.7	0.2	161	0
mask-095	ID_25	Inner	1	2017/5/17	3:00	13.1	0.2	89.5	0.6
mask-096	ID_25	Outer	3	2017/5/17	1:00	LDL	-	2.75	0.13
mask-097	ID_25	Inner	3	2017/5/17	1:00	LDL	-	0.923	0.087
mask-098	ID_25	Outer	4	2017/5/17	1:00	0.348	0.062	2.42	0.13
mask-099	ID_25	Inner	4	2017/5/17	1:00	LDL	-	1.81	0.11
mask-100	ID_26	Outer	1	2017/5/25	4:00	2.00	0.09	13.8	0.3
mask-101	ID_26	Inner	1	2017/5/25	4:00	1.55	0.08	10.5	0.2
mask-102	ID_26	Outer	2	2017/5/25	4:00	3.55	0.10	23.9	0.3
mask-103	ID_26	Inner	2	2017/5/25	4:00	2.75	0.10	18.0	0.3
mask-104	ID_26	Outer	3	2017/5/25	4:30	2.12	0.09	14.5	0.3
mask-105	ID_26	Inner	3	2017/5/25	4:30	1.95	0.09	13.8	0.3
mask-106	ID_26	Outer	5	2017/5/25	4:00	1.50	0.08	9.95	0.22
mask-107	ID_26	Inner	5	2017/5/25	4:00	1.22	0.07	9.03	0.21
mask-108	ID_27	Outer	1	2017/5/30	4:10	1.28	0.08	8.78	0.21
mask-109	ID_27	Inner	1	2017/5/30	4:10	1.54	0.08	9.97	0.22
mask-110	ID_27	Outer	3	2017/5/30	4:50	0.474	0.053	3.56	0.14
mask-111	ID_27	Inner	3	2017/5/30	4:50	0.362	0.049	2.13	0.11
mask-112	ID_27	Outer	5	2017/5/30	4:50	1.31	0.07	7.87	0.20
mask-113	ID_27	Inner	5	2017/5/30	4:50	0.889	0.064	6.13	0.18
mask-114	ID_28	Outer	1	2017/6/15	4:10	1.65	0.09	12.2	0.2
mask-115	ID_28	Inner	1	2017/6/15	4:10	2.02	0.08	14.6	0.3
mask-116	ID_28	Outer	3	2017/6/15	5:10	0.111	0.075	7.35	0.19
mask-117	ID_28	Inner	3	2017/6/15	5:10	3.63	0.11	25.4	0.3
mask-118	ID_28	Outer	4	2017/6/15	5:10	1.95	0.09	13.5	0.3
mask-119	ID_28	Inner	4	2017/6/15	5:10	1.98	0.09	14.9	0.3
mask-120	ID_01, 02	Outer	3	2017/7/11	6:00	9.27	0.16	63.5	0.5
mask-121	ID_01, 02	Inner	3	2017/7/11	6:00	9.39	0.16	65.8	0.6

mask-122	ID_01, 02	Outer	5	2017/7/11	6:00	15.3	0.2	108	1
mask-123	ID_01, 02	Inner	5	2017/7/11	6:00	14.6	0.2	102	1
mask-124	ID_02	Outer	1	2017/7/11	1:15	5.60	0.13	3.88	0.43
mask-125	ID_02	Inner	1	2017/7/11	1:15	4.86	0.12	35.0	0.4
mask-126	ID_01	Outer	1	2017/7/11	1:40	12.2	0.2	84.4	0.6
mask-127	ID_01	Inner	1	2017/7/11	1:40	18.8	0.2	131	1
mask-128	ID_29	Single	1	2017/7/12	2:00	LDL	-	0.869	0.081
mask-129	ID_29	Outer	3	2017/7/12	6:30	0.575	0.057	4.07	0.15
mask-130	ID_29	Inner	3	2017/7/12	6:30	0.813	0.061	6.01	0.19
mask-131	ID_29	Outer	5	2017/7/12	6:30	2.99	0.10	21.6	0.3
mask-132	ID_29	Inner	5	2017/7/12	6:30	1.86	0.08	13.2	0.3
mask-133	ID_29	Outer	1	2017/7/12	2:30	2.57	0.09	21.5	0.3
mask-134	ID_29	Inner	1	2017/7/12	2:30	2.20	0.09	16.0	0.3
mask-135	ID_30,31	Outer	1	2017/8/29	4:00	LDL	-	LDL	-
mask-136	ID_30,31	Inner	1	2017/8/29	4:00	LDL	-	LDL	-
mask-137	ID_30,31	Outer	2	2017/8/29	4:00	LDL	-	0.543	0.073
mask-138	ID_30,31	Inner	2	2017/8/29	4:00	LDL	-	0.964	0.085
mask-139	ID_30	Outer	4	2017/8/29	3:35	0.770	0.061	5.48	0.17
mask-140	ID_30	Inner	4	2017/8/29	3:35	LDL	-	1.00	0.08
mask-141	ID_30	Outer	1	2017/8/29	3:00	2.13	0.09	16.7	0.3
mask-142	ID_30	Inner	1	2017/8/29	3:00	3.08	0.11	24.2	0.3
mask-143	ID_30	Outer	2	2017/8/29	3:00	2.97	0.10	21.3	0.3
mask-144	ID_30	Inner	2	2017/8/29	3:00	2.71	0.11	18.7	0.3
mask-145	ID_30	Outer	3	2017/8/29	3:30	LDL	-	1.44	0.10
mask-146	ID_30	Inner	3	2017/8/29	3:30	0.592	0.064	4.47	0.16
mask-147	ID_31	Outer	4	2017/8/29	2:00	LDL	-	0.661	0.071
mask-148	ID_31	Inner	4	2017/8/29	2:00	LDL	-	0.677	0.077
mask-149	ID_31	Outer	1	2017/8/30	2:00	0.901	0.068	6.32	0.18
mask-150	ID_31	Inner	1	2017/8/30	2:30	0.560	0.051	4.59	0.16
mask-151	ID_31	Outer	2	2017/8/30	2:00	1.51	0.09	10.6	0.2
mask-152	ID_31	Inner	2	2017/8/30	2:30	2.03	0.09	14.3	0.3
mask-153	ID_31	Outer	3	2017/8/30	2:00	LDL	-	0.563	0.077
mask-154	ID_31	Inner	3	2017/8/30	2:00	LDL	-	0.576	0.073
mask-155	ID_32	Outer	3	2017/8/29	3:30	LDL	-	1.02	0.08
mask-156	ID_32	Inner	3	2017/8/29	3:30	LDL	-	0.776	0.077
mask-157	ID_32	Outer	4	2017/8/30	4:00	LDL	-	0.534	0.069
mask-158	ID_32	Inner	4	2017/8/30	4:00	LDL	-	0.354	0.061
mask-159	ID_32	Outer	1	2017/8/30	1:30	LDL	-	1.87	0.11
mask-160	ID_32	Inner	1	2017/8/30	1:30	0.321	0.076	2.30	0.12
mask-161	ID_32	Outer	2	2017/8/30	1:30	0.653	0.056	4.95	0.16
mask-162	ID_32	Inner	2	2017/8/30	1:30	0.793	0.066	5.61	0.17
mask-163	ID_32	Outer	4	2017/8/30	1:10	LDL	-	0.667	0.085
mask-164	ID_32	Inner	4	2017/8/30	1:10	LDL	-	0.441	0.078
mask-165	ID_33	Inner	1	2017/9/27	2:00	1.92	0.09	14.2	0.3
mask-166	ID_33	Outer	1	2017/9/27	2:00	1.78	0.09	15.0	0.3
mask-167	ID_33	Single	2	2017/9/27	2:00	3.56	0.12	29.8	0.4
mask-168	ID_34	Outer	1	2017/9/27	1:00	0.433	0.531	3.96	0.16
mask-169	ID_34	Inner	1	2017/9/27	1:00	0.300	0.071	2.17	0.13
mask-170	ID_34	Single	2	2017/9/27	1:00	1.68	0.09	14.0	0.3
mask-171	ID_35	Outer	1	2017/9/26	2:50	6.65	0.03	55.4	0.5
mask-172	ID_35	Inner	1	2017/9/26	2:50	1.78	0.09	13.5	0.3
mask-173	ID_35	Single	1	2017/9/26	1:00	LDL	-	0.408	0.070
mask-174	ID_35	Single	2	2017/9/26	4:05	6.66	0.15	53.3	0.5
mask-175	ID_33,34	Single	1	2017/9/26	1:00	LDL	-	0.449	0.073
mask-176	ID_33,34	Single	2	2017/9/26	1:00	LDL	-	1.23	0.09
mask-177	ID_33,34,35	Outer	3	2017/9/26	8:30	LDL	-	1.90	0.11
mask-178	ID_33,34,35	Inner	3	2017/9/26	8:30	0.767	0.075	6.66	0.19
mask-179	ID_33,34,35	Outer	5	2017/9/26	8:30	LDL	-	2.09	0.13
mask-180	ID_33,34,35	Inner	5	2017/9/26	8:30	0.510	0.065	4.62	0.16
mask-181	ID_33,34,35	Outer	5	2017/9/27	6:00	0.339	0.064	2.43	0.12
mask-182	ID_33,34,35	Inner	5	2017/9/27	6:00	LDL	-	1.18	0.09
mask-183	ID_33,34,35	Outer	3	2017/9/27	7:00	LDL	-	1.67	0.11

mask-184	ID_33,34,35	Inner	3	2017/9/27	7:00	LDL	-	1.12	0.09
mask-185	ID_36,37	Single	1	2017/10/25	2:00	LDL	-	0.796	0.078
mask-186	ID_36,37	Single	3	2017/10/25	4:00	0.330	0.059	1.70	0.11
mask-187	ID_36	Outer	5	2017/10/25	-	0.526	0.083	3.11	0.14
mask-188	ID_36	Inner	5	2017/10/25	-	0.316	0.072	1.66	0.11
mask-189	ID_36	Single	1	2017/10/25	1:20	1.69	0.09	13.6	0.3
mask-190	ID_37	Outer	5	2017/10/26	5:30	LDL	-	1.12	0.09
mask-191	ID_37	Inner	5	2017/10/26	5:30	LDL	-	0.583	0.078
mask-192	ID_37	Single	1	2017/10/26	1:30	2.04	0.10	17.8	0.3
mask-193	ID_37	Single	3	2017/10/26	5:30	0.938	0.074	7.50	0.20
mask-194	ID_38	Single	1	2017/10/26	1:30	LDL	-	1.46	0.10
mask-195	ID_38	Single	1	2017/10/26	1:40	3.95	0.12	33.5	0.4
mask-196	ID_39	Single	1	2017/11/8	1:30	LDL	-	0.364	0.058
mask-197	ID_39	Single	4	2017/11/8	6:30	2.11	0.09	16.6	0.3
mask-198	ID_39	Single	3	2017/11/8	5:30	0.458	0.064	4.81	0.16
mask-199	ID_39	Single	1	2017/11/8	2:00	1.77	0.10	15.4	0.3
mask-200	ID_40A	Single	1	2017/12/18	1:20	0.823	0.068	6.02	0.18
mask-201	ID_40A	Outer	2	2017/12/18	-	1.19	0.07	8.53	0.21
mask-202	ID_40A	Inner	2	2017/12/18	-	0.805	0.067	7.13	0.19
mask-203	ID_40B	Single	1	2017/12/18	1:30	0.817	0.067	6.52	0.18
mask-204	ID_40B	Outer	2	2017/12/18	-	0.562	0.068	5.02	0.17
mask-205	ID_40B	Inner	2	2017/12/18	-	0.615	0.068	5.31	0.17
mask-206	ID_40A, 40B	Single	3	2017/12/18	9:00	LDL	-	4.06	0.15
mask-207	ID_40A, 40B	Single	5	2017/12/18	-	LDL	-	1.25	0.09
mask-208	ID_41B	Single	1	2017/12/19	3:30	0.733	0.079	6.46	0.18
mask-209	ID_41B	Single	2	2017/12/19	3:30	0.957	0.074	7.65	0.20
mask-210	ID_42A	Single	1	2017/12/19	2:30	0.724	0.069	6.07	0.18
mask-211	ID_42A	Single	2	2017/12/19	2:30	1.30	0.08	10.0	0.2
mask-212	ID_41B,42A	Single	5	2017/12/19	-	LDL	-	0.986	0.087
mask-213	ID_45	Single	1	2018/4/23	2:00	LDL	-	LDL	-
mask-214	ID_45	Single	2	2018/4/23	2:00	LDL	-	0.418	0.064
mask-215	ID_45	Single	3	2018/4/23	3:10	LDL	-	0.495	0.069
mask-216	ID_45	Single	3	2018/4/23	3:40	LDL	-	1.17	0.09
mask-217	ID_45	Single	6	2018/4/23	3:40	LDL	-	1.46	0.10
mask-218	ID_46	Single	6	2018/4/23	3:10	0.374	0.052	3.49	0.14
mask-219	ID_46	N95 respirator	1	2018/4/23	1:50	LDL	-	0.760	0.044
mask-220	ID_46	N95 respirator	2	2018/4/23	1:50	0.147	0.028	2.04	0.07
mask-221	ID_46	Single	1	2018/4/24	1:40	LDL	-	LDL	-
mask-222	ID_46	Single	2	2018/4/24	1:55	LDL	-	1.45	0.10
mask-223	ID_46	Single	3	2018/4/24	2:00	LDL	-	0.343	0.061
mask-224	ID_46	Single	6	2018/4/24	2:00	LDL	-	0.801	0.085
mask-225	ID_46	N95 respirator	1	2018/4/24	1:30	LDL	-	LDL	-
mask-226	ID_46	N95 respirator	2	2018/4/24	1:30	0.300	0.040	3.01	0.08
mask-227	ID_46	Single	3	2018/4/24	2:00	LDL	-	0.591	0.072
mask-228	ID_46	Single	6	2018/4/24	2:00	LDL	-	1.45	0.10
mask-229	ID_47	Single	1	2018/5/8	2:00	LDL	-	LDL	-
mask-230	ID_47	Single	2	2018/5/8	2:10	LDL	-	LDL	-
mask-231	ID_47	Single	3	2018/5/8	3:00	LDL	-	LDL	-
mask-232	ID_47	Single	6	2018/5/8	3:00	LDL	-	LDL	-
mask-233	ID_47	N95 respirator	1	2018/5/8	1:10	LDL	-	0.406	0.034
mask-234	ID_47	N95 respirator	2	2018/5/8	1:10	0.236	0.027	2.27	0.07
mask-235	ID_47	Single	3	2018/5/8	2:30	LDL	-	0.431	0.071
mask-236	ID_47	Single	6	2018/5/8	2:30	LDL	-	LDL	-
mask-237	ID_48	Single	1	2018/5/30	2:00	LDL	-	LDL	-
mask-238	ID_48	Single	2	2018/5/30	2:00	LDL	-	0.917	0.087
mask-239	ID_48	Single	6	2018/5/30	2:00	LDL	-	2.57	0.12
mask-240	ID_48	Single	6	2018/5/30	2:00	2.13	0.10	18.4	0.3
mask-241	ID_48	Single	3	2018/5/30	5:40	1.38	0.09	12.1	0.2
mask-242	ID_48	N95 respirator	1	2018/5/30	2:00	1.37	0.05	12.9	0.2
mask-243	ID_48	N95 respirator	2	2018/5/30	2:00	4.15	0.07	39.3	0.3
mask-244	ID_49	Single	1	2018/7/9	1:30	LDL	-	LDL	-
mask-245	ID_49	Single	2	2018/7/9	1:25	0.407	0.125	2.95	0.13

mask-246	ID_49	Single	6	2018/7/9	1:50	0.871	0.072	8.34	0.21
mask-247	ID_49	N95 respirator	1	2018/7/9	1:50	0.521	0.032	4.61	0.10
mask-248	ID_49	N95 respirator	2	2018/7/9	1:55	1.24	0.04	12.2	0.2
mask-249	ID_49	Single	3	2018/7/9	4:15	0.905	0.074	8.72	0.21
mask-250	ID_49	Single	6	2018/7/9	1:30	0.413	0.069	4.13	0.15
mask-251	ID_50	Single	1	2018/9/26	2:00	LDL	-	LDL	-
mask-252	ID_50	Single	2	2018/9/26	2:00	LDL	-	2.66	0.14
mask-253	ID_50	Single	6	2018/9/26	1:35	1.03	0.07	8.87	0.21
mask-254	ID_50	Outer	1	2018/9/26	2:15	0.604	0.062	5.75	0.18
mask-255	ID_50	Inner	1	2018/9/26	2:15	0.579	0.062	4.65	0.16
mask-256	ID_50	N95 respirator	2	2018/9/26	2:00	0.880	0.036	8.69	0.13
mask-257	ID_50	Single	6	2018/9/26	1:05	0.376	0.063	3.81	0.15
mask-258	ID_50,51	Single	3	2018/9/26	4:20	0.337	0.668	2.50	0.12
mask-259	ID_51	Single	6	2018/9/26	1:00	LDL	-	1.66	0.10
mask-260	ID_51	Single	1	2018/9/27	2:00	LDL	-	LDL	-
mask-261	ID_51	Single	2	2018/9/27	2:00	LDL	-	0.952	0.085
mask-262	ID_51	Single	6	2018/9/27	2:15	0.396	0.006	2.84	0.13
mask-263	ID_51	Outer	1	2018/9/27	2:00	0.960	0.074	10.9	0.2
mask-264	ID_51	Inner	1	2018/9/27	2:00	LDL	-	2.24	0.12
mask-265	ID_51	N95 respirator	2	2018/9/27	2:00	1.01	0.05	9.83	0.14
mask-266	ID_52	Single	1	2018/10/11	1:50	LDL	-	1.50	0.10
mask-267	ID_52	Single	6	2018/10/11	4:00	LDL	-	0.674	0.073
mask-268	ID_53	Single	1	2018/10/11	1:40	LDL	-	LDL	-
mask-269	ID_53	N95 respirator	2	2018/10/11	1:50	0.352	0.027	4.02	0.09
mask-270	ID_54	N95 respirator	2	2018/10/12	1:40	LDL	-	0.688	0.043
mask-271	ID_54	Single	1	2018/11/14	1:50	LDL	-	0.880	0.081
mask-272	ID_54	N95 respirator	2	2018/11/14	1:40	0.155	0.020	2.18	0.07
mask-273	ID_55	Single	1	2018/11/15	1:10	LDL	-	0.760	0.083
mask-274	ID_55	N95 respirator	2	2018/11/15	1:20	0.100	0.018	0.976	0.049
mask-275	ID_56	Single	1	2018/11/30	1:45	1.30	0.11	14.1	0.3
mask-276	ID_56	N95 respirator	2	2018/11/30	1:45	2.18	0.05	24.3	0.2
mask-277	ID_58	Single	1	2018/12/20	1:30	LDL	-	0.490	0.062
mask-278	ID_58	N95 respirator	2	2018/12/20	1:35	LDL	-	0.337	0.035
mask-279	ID_59	Single	1	2018/12/21	0:20	LDL	-	LDL	-
mask-280	ID_59	N95 respirator	2	2018/12/21	1:40	LDL	-	0.683	0.042
mask-281	ID_60	Single	1	2019/1/23	3:30	0.919	0.157	4.88	0.16
mask-282	ID_60	Single	2	2019/1/23	2:30	5.72	0.18	60.2	0.5
mask-283	ID_61	Single	1	2019/1/24	1:40	3.41	0.19	34.4	0.4
mask-284	ID_61	Single	2	2019/1/24	1:40	17.9	0.3	196	1

LDL: Lower the detection limit.

Supplementary Table S2 Percentage of CsMPs found in each class for ^{137}Cs radioactivity in the masks.

Class for radioactivity of ^{137}Cs of each mask [Bq]	Number of masks	Number of masks for which CsMP was found	Ratio(%)
over 1000	1	1	100.0
100 - 1000	12	3	25.0
10 - 100	110	21	19.1
1 - 10	106	3	2.8
0.1 - 1	39	0	0.0
0 - 0.1	16	0	0.0
Total	284	28	

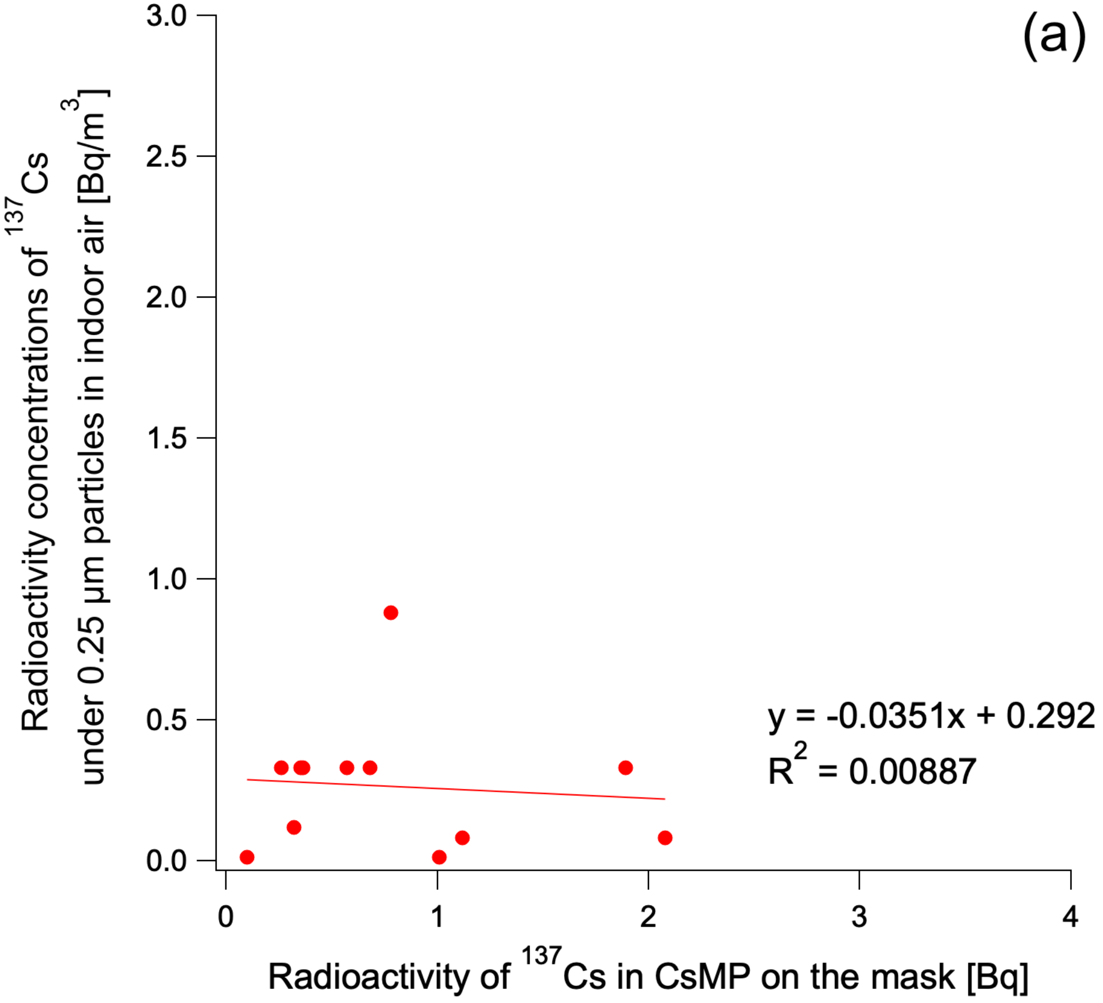

Supplementary Figure S1 (a) Relationship between the radioactivity of ^{137}Cs in CsMP in the mask (excluding mask-012, where non-spherical CsMPs were found) and the radioactivity of under 0.25 μm particles in indoor air reported by Yoshida-Ohuchi and Shinohara (2020). Radioactivity is decay corrected to March 11, 2011.

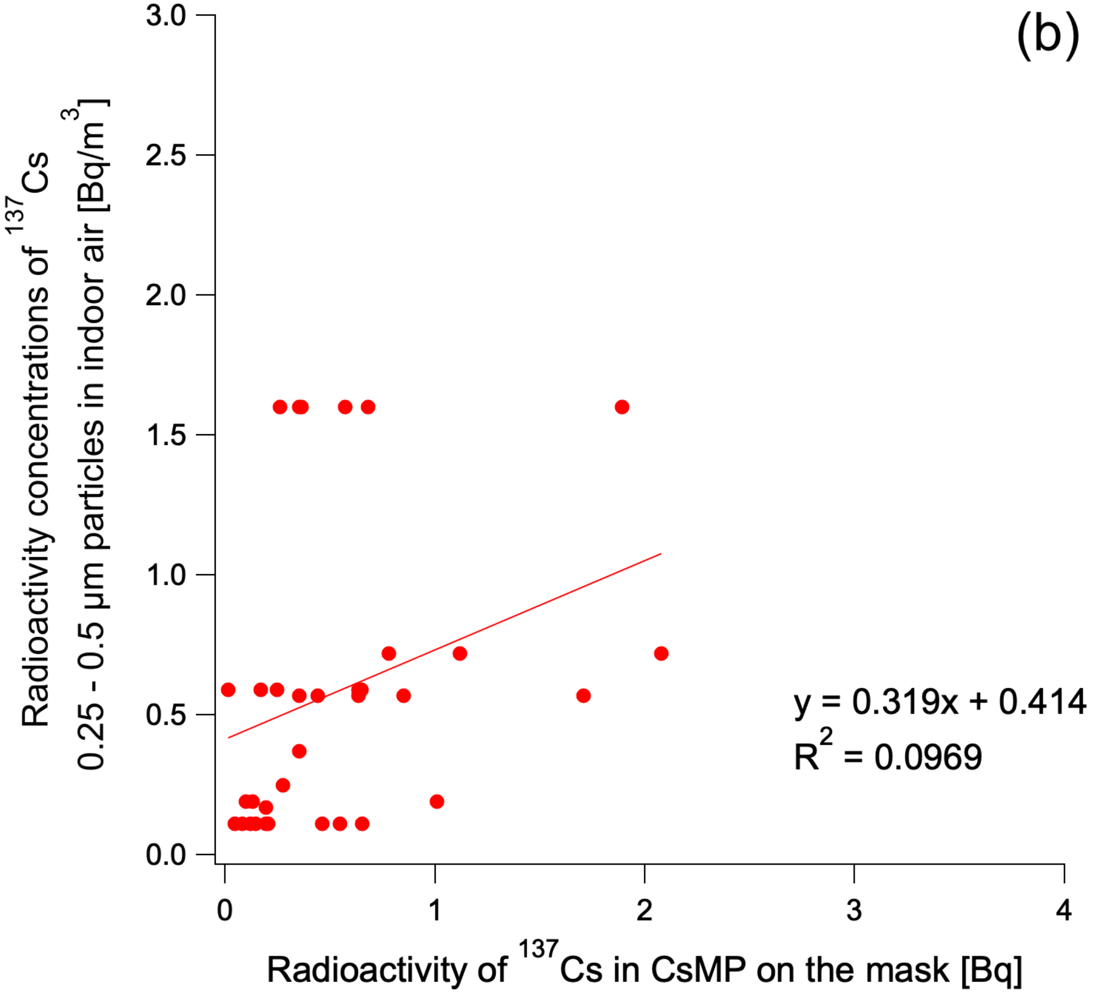

Supplementary Figure S1 (b) Relationship between the radioactivity of ^{137}Cs in CsMP in the mask (excluding mask-012, where non-spherical CsMPs were found) and the radioactivity of 0.25–0.5 µm particles in indoor air reported by Yoshida-Ohuchi and Shinohara (2020). Radioactivity is decay corrected to March 11, 2011.

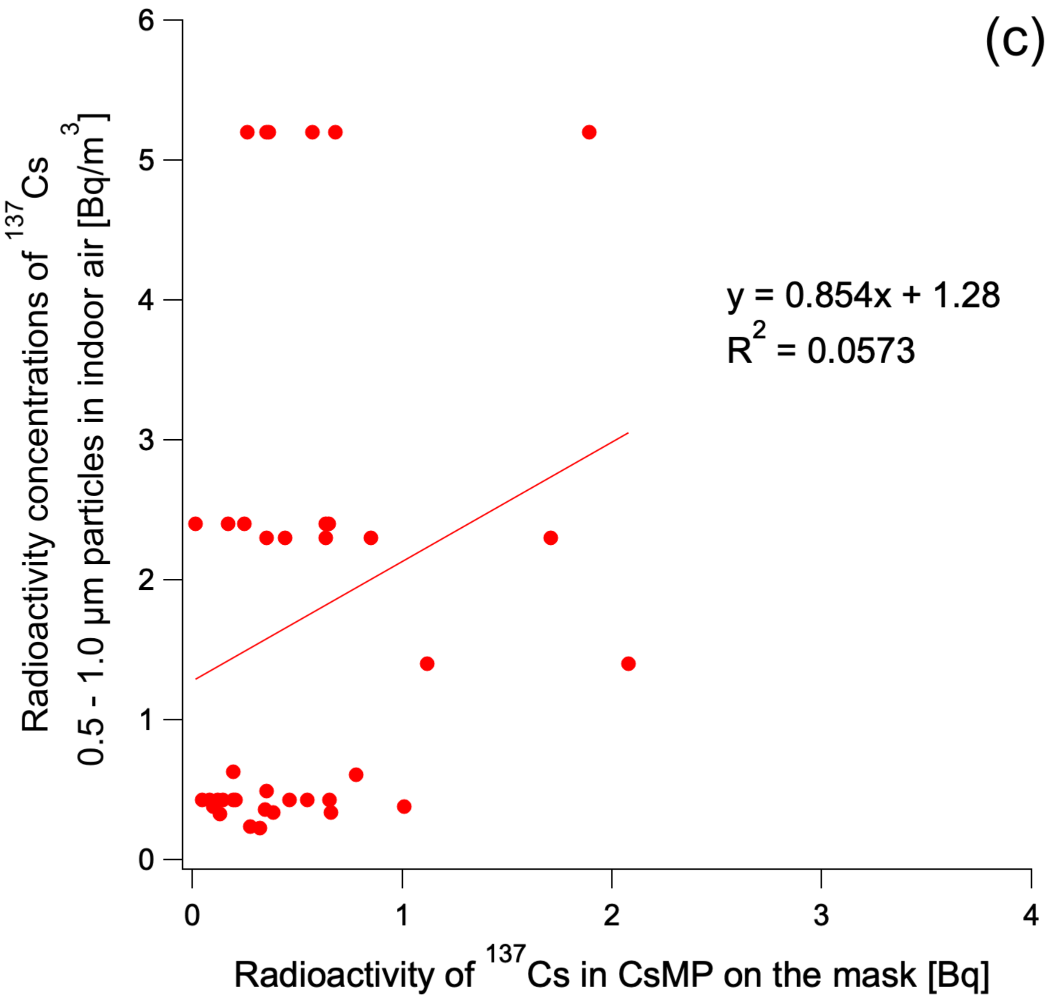

Supplementary Figure S1 (c) Relationship between the radioactivity of ^{137}Cs in CsMP in the mask (excluding mask-012, where non-spherical CsMPs were found) and the radioactivity of 0.5–1.0 µm particles in indoor air reported by Yoshida-Ohuchi and Shinohara (2020). Radioactivity is decay corrected to March 11, 2011.

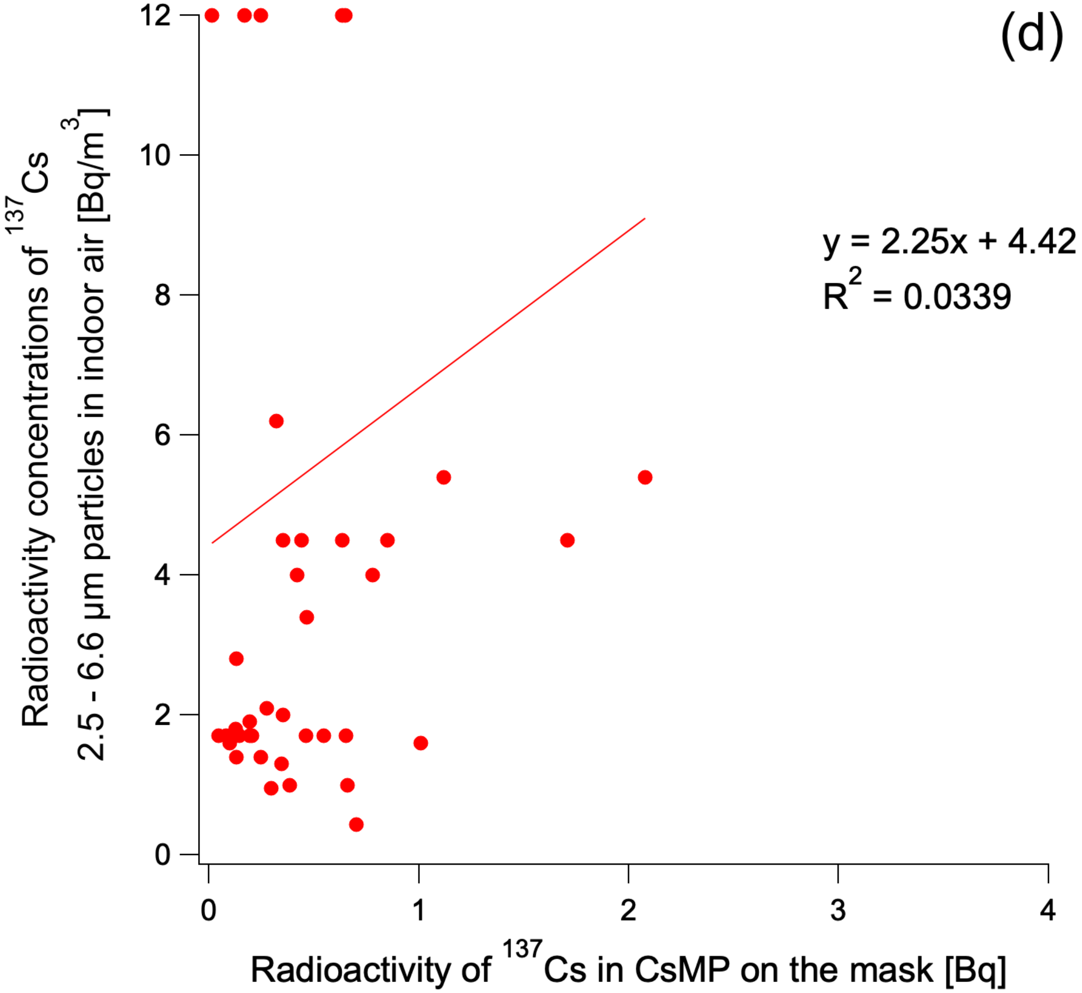

Supplementary Figure S1 (d) Relationship between the radioactivity of ^{137}Cs in CsMP in the mask (excluding mask-012, where non-spherical CsMPs were found) and the radioactivity of 2.5–6.6 µm particles in indoor air reported by Yoshida-Ohuchi and Shinohara (2020). Radioactivity is decay corrected to March 11, 2011.

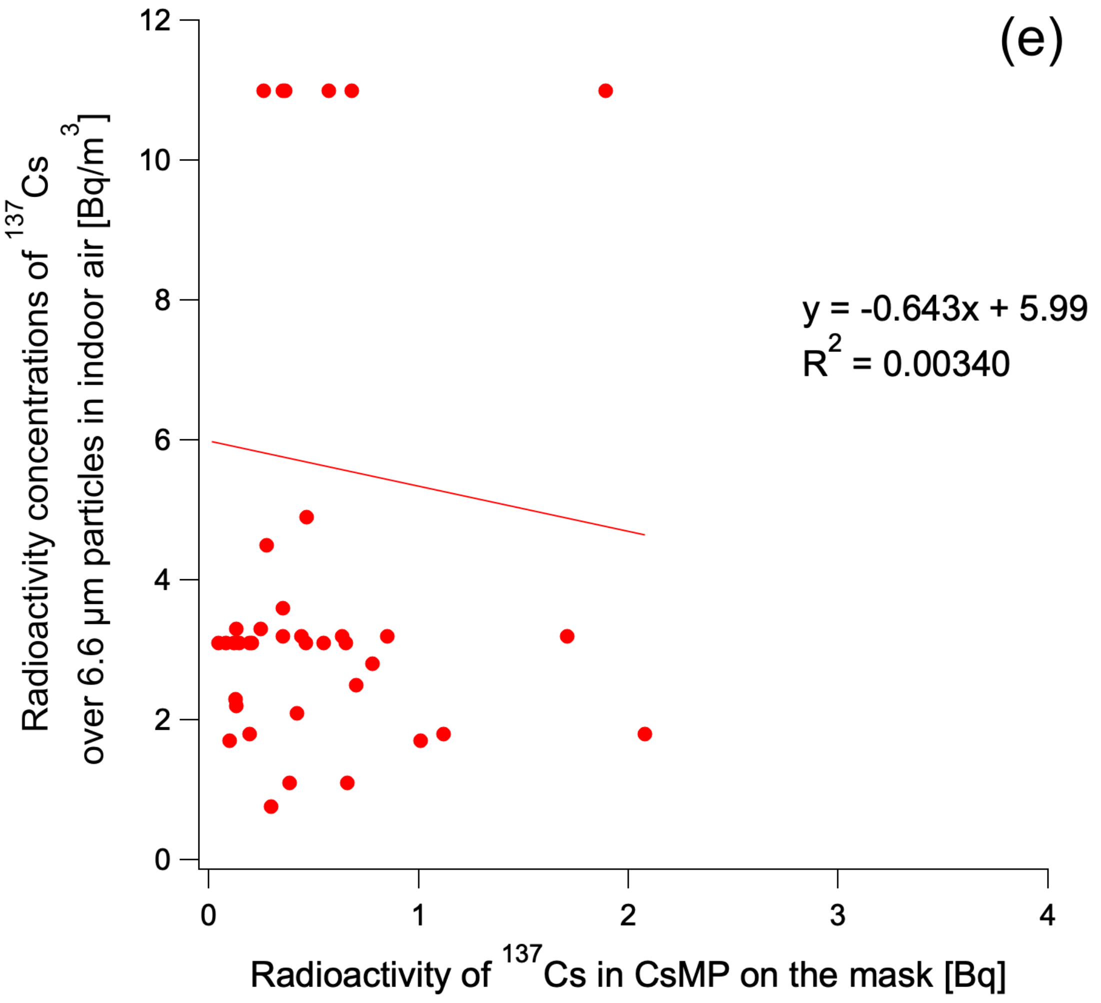

Supplementary Figure S1 (e) Relationship between the radioactivity of ^{137}Cs in CsMP in the mask (excluding mask-012, where non-spherical CsMPs were found) and the radioactivity of over 6.6 μm particles in indoor air reported by Yoshida-Ohuchi and Shinohara (2020). Radioactivity is decay corrected to March 11, 2011.

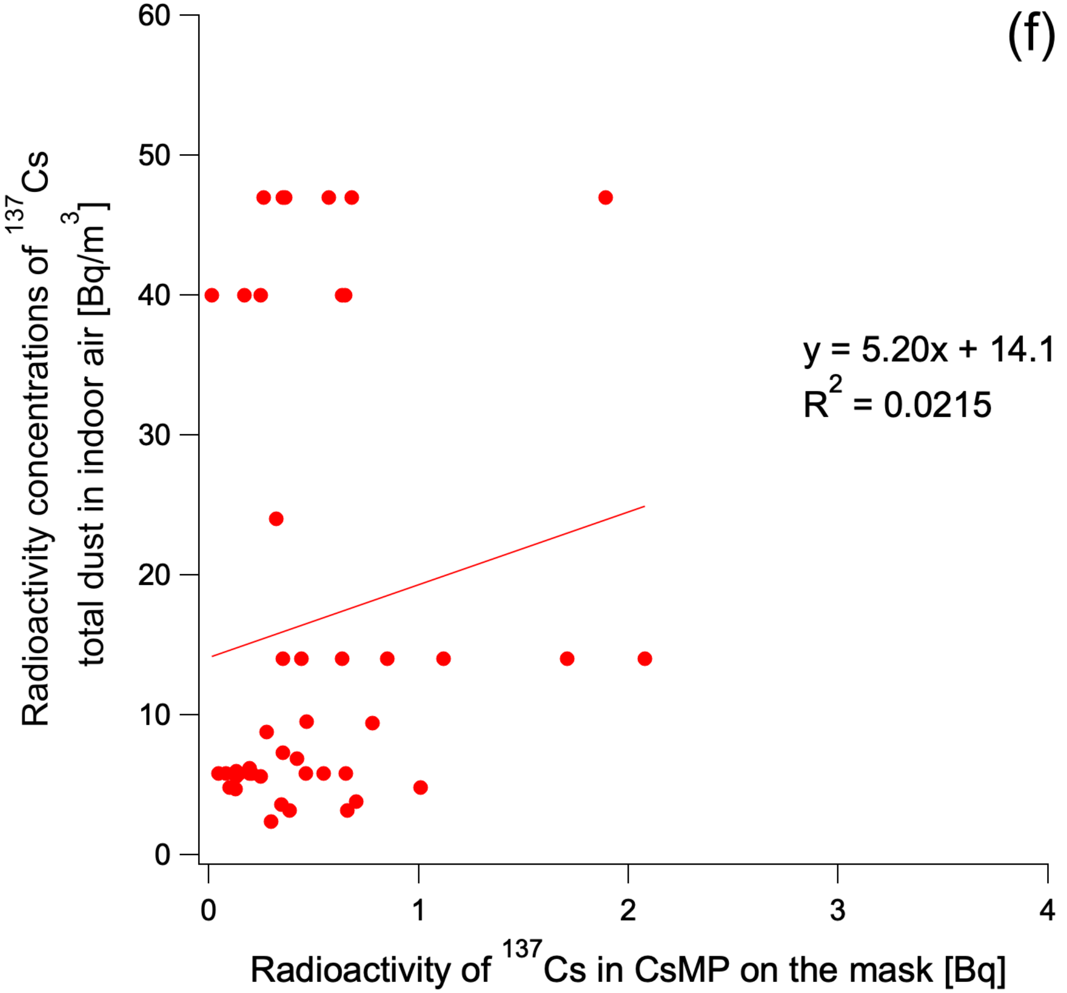

Supplementary Figure S1 (f) Relationship between the radioactivity of ^{137}Cs in CsMP in the mask (excluding mask-012, where non-spherical CsMPs were found) and the radioactivity of total dust in indoor air reported by Yoshida-Ohuchi and Shinohara (2020). Radioactivity is decay corrected to March 11, 2011.

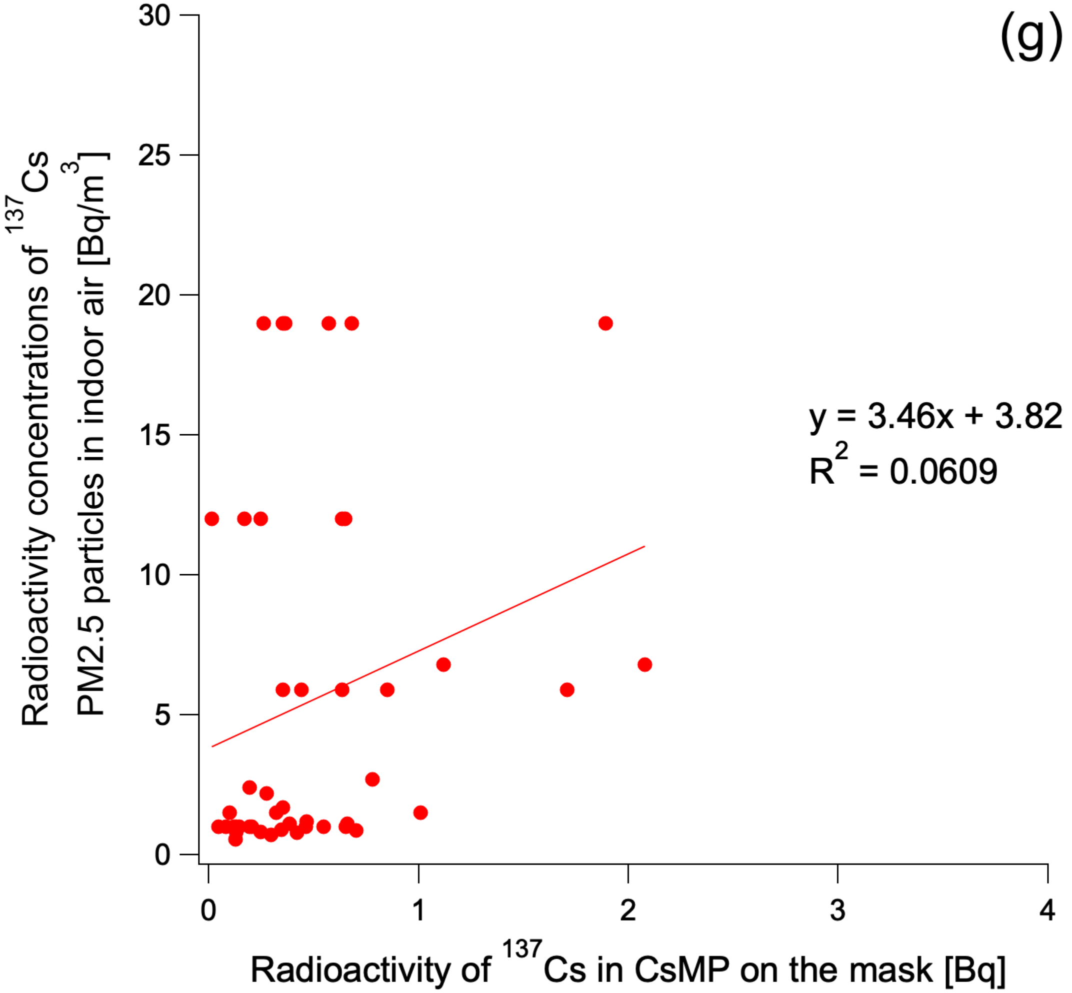

Supplementary Figure S1 (g) Relationship between the radioactivity of ^{137}Cs in CsMP in the mask (excluding mask-012, where non-spherical CsMPs were found) and the radioactivity of PM2.5 particles in indoor air reported by Yoshida-Ohuchi and Shinohara (2020). Radioactivity is decay corrected to March 11, 2011.
